# A novel multiplexed biomarker panel reveals key molecular pathways in progressive multiple sclerosis

**DOI:** 10.1093/braincomms/fcag142

**Published:** 2026-04-17

**Authors:** Christina N Heiss, Lenka Novakova, Markus Axelsson, Kübra Tan, Ilaria Pola, Wiebke Traichel, Ida Pesämaa, Magnus Johnsson, Sofia Sandgren, Clas Malmeström, Andrea L Benedet, Henrik Zetterberg, Jan N Lycke, Stefanie Fruhwürth, Igal Rosenstein

**Affiliations:** Department of Psychiatry and Neurochemistry, Institute of Neuroscience and Physiology, University of Gothenburg, Gothenburg 41345, Sweden; Department of Clinical Neuroscience, Institute of Neuroscience and Physiology at Sahlgrenska Academy, University of Gothenburg, Gothenburg 41345, Sweden; Department of Neurology, Region Västra Götaland, Sahlgrenska University Hospital, Gothenburg 41345, Sweden; Department of Clinical Neuroscience, Institute of Neuroscience and Physiology at Sahlgrenska Academy, University of Gothenburg, Gothenburg 41345, Sweden; Department of Neurology, Region Västra Götaland, Sahlgrenska University Hospital, Gothenburg 41345, Sweden; Department of Psychiatry and Neurochemistry, Institute of Neuroscience and Physiology, University of Gothenburg, Gothenburg 41345, Sweden; Department of Psychiatry and Neurochemistry, Institute of Neuroscience and Physiology, University of Gothenburg, Gothenburg 41345, Sweden; Department of Psychiatry and Neurochemistry, Institute of Neuroscience and Physiology, University of Gothenburg, Gothenburg 41345, Sweden; Department of Psychiatry and Neurochemistry, Institute of Neuroscience and Physiology, University of Gothenburg, Gothenburg 41345, Sweden; Department of Clinical Neuroscience, Institute of Neuroscience and Physiology at Sahlgrenska Academy, University of Gothenburg, Gothenburg 41345, Sweden; Department of Neurology, Region Västra Götaland, Sahlgrenska University Hospital, Gothenburg 41345, Sweden; Department of Clinical Neuroscience, Institute of Neuroscience and Physiology at Sahlgrenska Academy, University of Gothenburg, Gothenburg 41345, Sweden; Department of Neurology, Region Västra Götaland, Sahlgrenska University Hospital, Gothenburg 41345, Sweden; Department of Clinical Neuroscience, Institute of Neuroscience and Physiology at Sahlgrenska Academy, University of Gothenburg, Gothenburg 41345, Sweden; Department of Neurology, Region Västra Götaland, Sahlgrenska University Hospital, Gothenburg 41345, Sweden; Department of Psychiatry and Neurochemistry, Institute of Neuroscience and Physiology, University of Gothenburg, Gothenburg 41345, Sweden; Department of Psychiatry and Neurochemistry, Institute of Neuroscience and Physiology, University of Gothenburg, Gothenburg 41345, Sweden; Clinical Neurochemistry Laboratory, Sahlgrenska University Hospital, Mölndal 43139, Sweden; UK Dementia Research Institute Fluid Biomarkers laboratory at UCL, London NW1 3BT, UK; Department of Neurodegenerative Disease, UCL Queen Square Institute of Neurology, London WC1N 3BG, UK; Hong Kong Centre for Neurodegenerative Diseases, Hong Kong University of Science and Technology, Hong Kong 999077, China; Wisconsin Alzheimer’s Disease Research Center, University of Wisconsin School of Medicine and Public Health, University of Wisconsin-Madison, Madison, WI 53792, USA; Centre for Brain Research, Indian Institute of Science, Bangalore 560012, India; Department of Clinical Neuroscience, Institute of Neuroscience and Physiology at Sahlgrenska Academy, University of Gothenburg, Gothenburg 41345, Sweden; Department of Neurology, Region Västra Götaland, Sahlgrenska University Hospital, Gothenburg 41345, Sweden; Department of Psychiatry and Neurochemistry, Institute of Neuroscience and Physiology, University of Gothenburg, Gothenburg 41345, Sweden; Department of Clinical Neuroscience, Institute of Neuroscience and Physiology at Sahlgrenska Academy, University of Gothenburg, Gothenburg 41345, Sweden; Department of Neurology, Region Västra Götaland, Sahlgrenska University Hospital, Gothenburg 41345, Sweden

**Keywords:** biomarkers, neuroinflammation, multiplex immunoassay, stem cell factor, c-kit signalling

## Abstract

Progressive multiple sclerosis is characterized by gradual neurological decline, often occurring independently of relapses or MRI activity—a phenomenon known as progression independent of relapse and MRI activity (PIRMA). Despite the effectiveness of disease-modifying therapies in controlling inflammatory activity, identifying individuals at risk of PIRMA remains an unmet clinical need. The objective of this exploratory study was to identify biomarkers and underlying molecular pathways associated with multiple sclerosis progression and especially PIRMA. Using the NUcleic acid Linked Immuno-Sandwich Assay (NULISA) inflammatory panel, we quantified 250 immune-related proteins in CSF and plasma from 49 controls, 49 patients with early active relapsing-remitting multiple sclerosis (RRMS) and 33 patients with inactive progressive multiple sclerosis (iPMS). Longitudinal clinical data were used to define PIRMA and conversion to secondary progressive multiple sclerosis (SPMS). We identified distinct proteomic signatures in CSF of both RRMS and iPMS patients compared with controls, with no significant differences in plasma. Both were associated with elevated markers of adaptive immunity, while iPMS showed a shift towards innate immune markers. Among RRMS patients, low baseline CSF concentrations of KIT ligand (KITLG) predicted both conversion to SPMS and future PIRMA events. Receiver operating characteristic analysis demonstrated KITLG’s potential as a prognostic biomarker. Additionally, plasma concentrations of interleukin 1 beta and 36 gamma were elevated in RRMS patients who later developed SPMS. A model selection analysis identified a two-protein logistic regression model including interleukin 1 beta and interleukin 36 gamma as the best-performing combination (area under the curve = 0.990). Our findings reveal distinct immunological profiles across multiple sclerosis subtypes and identify KITLG as a promising biomarker for predicting disease progression and PIRMA. These results highlight potential targets for therapeutic intervention and demonstrate the utility of NULISA in uncovering novel molecular signatures in multiple sclerosis.

## Teaser

Heiss *et al*. use multiplexed profiling of cerebrospinal fluid and blood proteins to map inflammatory and neuroglial pathways across multiple sclerosis and demonstrate that a shift towards innate immune activation characterizes progressive disease. Their models show that low KIT ligand and specific blood proteins predict early transition to clinical worsening.

## Graphical Abstract

**Graphical Abstract:**
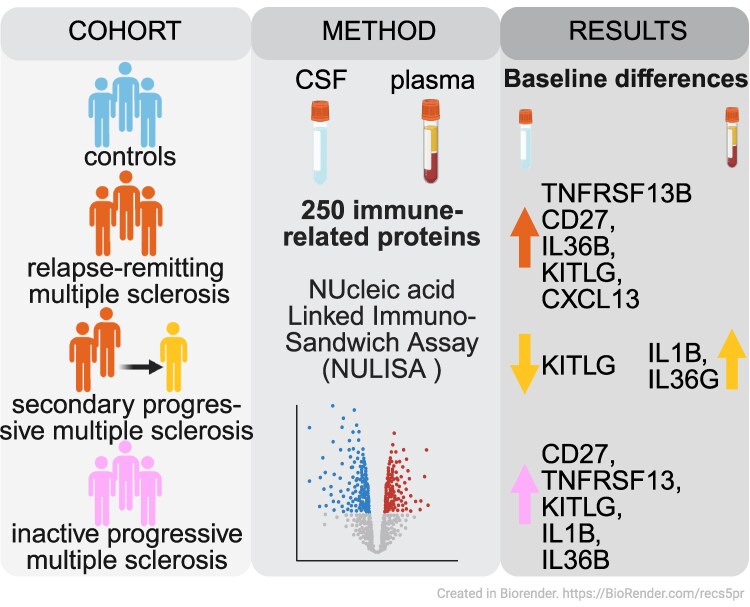

## Introduction

Multiple sclerosis (MS) has historically been characterized as a multifocal inflammatory and demyelinating disorder of the CNS. However, recent evidence challenges this conventional view, suggesting that MS is driven primarily by a smouldering process, with superimposed inflammatory disease activity.^1^ Clinically, this smouldering process is often identified as progression independent of relapse and MRI activity (PIRMA), a phenomenon where patients with relapsing-remitting MS (RRMS) experience gradual neurological deterioration despite the absence of inflammatory disease activity and even while receiving highly effective disease-modifying therapies (DMTs).^2^

The clinical course of MS has traditionally been classified into several distinct clinical subtypes or phenotypes: RRMS, secondary progressive MS (SPMS) and primary progressive MS (PPMS).^3^ Approximately 85% of MS patients initially exhibit a relapsing-remitting disease pattern, typically manifesting in young adults. The remaining 10–15% present with PPMS, characterized by a gradual and continuous accumulation of disability from disease onset. Over time, some patients with RRMS may transition to SPMS, marked by insidious worsening of disability independent of relapse activity.

The advent of highly effective DMTs has largely achieved the goal of halting relapses and preventing the formation of new lesions, as detected by MRI. However, the next major therapeutic frontier lies in addressing smouldering MS and its associated clinical phenotype, PIRMA. Emerging treatments such as Bruton tyrosine kinase inhibitors offer promise; notably, a recently published trial demonstrated a reduction in confirmed disability worsening (CDW) in patients with non-relapsing SPMS,^4^ although further studies are needed to confirm long-term efficacy and identify optimal treatment windows.^5^ Identifying smouldering processes early in the disease course and stratifying patients at higher risk of developing disability progression, despite controlled inflammatory activity, remains a critical unmet need.

While established biomarkers, such as the kappa free light chain index and serum glial fibrillary acidic protein (GFAP), have shown associations with PIRMA,^6,7^ much remains to be uncovered regarding the biological pathways driving disease progression in MS. Identifying potential drug targets involved in neuro-inflammatory pathways underlying smouldering MS is crucial for developing treatment options.

The aim of this study was to measure immune-related proteins using NUcleic acid Linked Immuno-Sandwich Assay (NULISA) in biofluids of patients with different clinical subtypes of MS to identify biomarkers of progressive MS and PIRMA. Here, we report distinct CSF proteomic profiles in RRMS and inactive PMS (iPMS) patients compared with controls, with no major differences in plasma. We found elevated markers of adaptive immunity (e.g. TNFRSF13B, CD27) in RRMS patients and a shift towards innate immune markers (e.g. CCL3, C1QA, IFNB1) in iPMS patients. Our exploratory findings point to possible distinctions in immune profiles across MS subtypes and raise the hypothesis that certain molecular signatures could be linked to progression.

## Methods

### Patients and samples

The neuro-biomarker biobank at the Department of Neurology, Sahlgrenska University Hospital (Sweden), includes blood and CSF samples collected during routine clinical investigations. From this biobank, we identified baseline samples (October 2010–October 2020) from:

- Patients with RRMS who remained stable (did not convert to SPMS during follow-up),
- Patients who transitioned from RRMS to SPMS during follow-up (SPMS conversion was defined as sustained disability progression for at least 1 year, independent of relapses, as assessed by the treating neurologist) and
- Patients with iPMS, defined as progressive-onset MS without preceding relapses or MRI evidence of inflammatory activity.^8,9^ Active PMS were not included due to overlapping inflammatory activity with RRMS, which may confound analyses focused on progression-related biology.

To obtain a representative, heterogeneous control group, we enrolled healthy donors (*n* = 14) and symptomatic controls (*n* = 35)—individuals presenting with MS-like symptoms but a negative diagnostic work-up for MS.^8,9^ Conditions with other inflammatory, infectious, or neurodegenerative aetiology were excluded.

Subgroup classification was based on the Swedish MS registry (SMSreg).^10^ All MS patients were newly diagnosed and treatment-naïve at inclusion and sampling. Collected clinical and demographic data of interest were: age, sex, disease course and duration, MRI measures [number of T2 lesions, contrast-enhancing lesions (CEL)], expanded disability status scale (EDSS)^11^ and exposure to DMTs. EDSS scores at baseline, and thereafter yearly throughout the whole follow-up, were retrieved from the SMSreg. All EDSS scores were obtained by experienced neurologists during routine clinical visits.

PIRMA was defined as a clinical CDW event that occurs independent of relapse/MRI activity (no clinical relapses or new/enlarging/contrast-enhancing MRI lesions 90 days before or after the event) that persists during the follow-up time.^12^ Sustained CDW was defined as a sustained increase in EDSS score along the study follow-up by ≥1.5, ≥1 and ≥0.5 if baseline EDSS was 0, 1.0–5.0 and ≥5.5, respectively. We used a roving baseline EDSS.^13^

MRI of the brain and spinal cord without and with gadolinium contrast was performed on 1.5 or 3.0 T machines, according to Swedish radiological guidelines.^14^ MRIs were obtained at baseline, about 6 months after baseline, and thereafter at least annually, and in addition, on the discretion of the treating physician in cases of suspected relapse.

### Ethical standards

All patients participated voluntarily in the study and provided written informed consent according to the Declaration of Helsinki. The study conformed to the Code of Ethics of the World Medical Association. The Swedish ethical review authority approved the study (Reference number: Dnr 2024-07656-01).

### NULISA proteomic analysis

Samples were handled according to the consensus protocol of the BioMS-EU network for CSF biomarker research in MS.^15^ Blood (EDTA tubes) and CSF (polypropylene tubes) samples were collected simultaneously, processed on-site to isolate plasma, carefully aliquoted in cryotubes and frozen at −80°C. No more than one freeze–thaw cycle occurred prior to assay.

CSF and plasma samples were analysed using the NULISA™ inflammation panel (Alamar Biosciences). Panel proteins were quantified through immunocomplex formation associated with DNA reporter molecule ligation. DNA reporter molecules were pooled, amplified by PCR, purified and sequenced using Illumina NextSeq 2000. The sequencing data were processed using the NULISAseq protocol as previously described.^16^ Data were rescaled and log_2_ transformed to obtain NULISA Protein Quantification (NPQ) units. The full protein list as well as validation data are accessible through the Alamar Biosciences website (https://alamarbio.com/products-and-services/nulisa-inflammation-panel/).

### Statistical analysis

Descriptive data are presented as median and interquartile range (IQR). The Kruskal–Wallis test with false discovery rate (FDR, two-stage linear step-up procedure of Benjamini, Krieger and Yekutieli), χ^2^ test and Fisher’s exact test were used for group comparisons, as appropriate.

To study associations between proteins in CSF and blood and MS disease progression, protein quantifications were compared between the different study subgroups (MS consisting of RRMS and iPMS versus controls, RRMS versus controls, iPMS versus controls, RRMS versus iPMS and stable RRMS versus RRMS patients who converted to SPMS). We investigated sustained PIRMA as a binary nominal dependent variable. All baseline group comparisons (i.e. controls versus MS, controls versus RRMS and controls versus iPMS) were adjusted for age and sex. As EDSS was strongly collinear with group assignment (RRMS versus iPMS), we opted not to include it to avoid multicollinearity. In contrast, models involving prognostic outcomes (RRMS->SPMS and PIRMA) were adjusted for age, sex and treatment category.

Initially, we performed a differential expression analysis to investigate protein-level differences between disease groups. Protein levels, already presented on log_2_ scale (raw NPQ values), were analysed without further transformation. For each protein, an ordinary least squares linear model was fitted with disease state as the primary predictor and age and sex included as covariates. Estimated coefficients represent differences in mean log_2_ NPQ levels (log_2_ fold changes). Significance was assessed using *P*-values adjusted for multiple comparisons using the Benjamini–Hochberg FDR. Results were illustrated using volcano plots displaying estimated effect sizes (differences in mean log_2_ NPQ values, log_2_ fold changes) against −log_10_  *P*-values. Differential expression significance was determined based on both nominal and FDR-adjusted *P*-value. Top-ranked proteins were additionally presented in heatmaps. Selective protein levels were visualized as boxplots across all disease groups, with statistical differences assessed using unpaired two-sided *t*-tests.

To investigate whether a panel of differentially expressed proteins (DEPs) may assist in distinguishing disease states at diagnosis, we performed binary logistic regression using top DEPs from each pairwise disease state comparison (e.g. iPMS versus RRMS). To reduce the number of proteins for logistic regression modelling, proteins were filtered after Pearson correlation with the binary disease outcome. Logistic regression models, including only protein predictors without adjustment for covariates such as age or sex, as control subjects were matched to cases for these variables, were ranked using the corrected Akaike Information Criterion (AICc). Predicted probabilities from the selected models were used to generate receiver operating characteristic (ROC) curves. Area under the curve (AUC) was used to assess classification performance, presented with 95% confidence intervals and *P*-values (DeLong method).

For the CSF samples, due to a larger sample size, we could perform model selection with model averaging across all models with ΔAICc ≤ 2 to achieve a robust composite model. This average model was used to calculate predicted probabilities and assess classification performance. The model was further tested by 10-fold cross-validation (CV) and compared based on AUCs of the generated ROC curves. The predictive ability of the average model was further compared with that of individual proteins using DeLong’s test for correlated ROC curves. For plasma samples and for PIRMA analysis, due to a smaller sample size, models within ΔAICc ≤ 4 were considered, and the single best-performing model was selected. Predicted probabilities were used again to generate ROC curves and calculate AUCs with 95% confidence intervals and *P*-values (DeLong’s method).

Next, we investigated the associations of the calculated cut-off values with the selected outcomes in multivariable Cox regression models. The adjusted hazard ratios, along with corresponding 95% CIs and *P*-values, were calculated. Based on previous investigations on prognostic factors,^17^ we adjusted the models for the following potential confounding covariates: age at sampling, sex, disease duration prior to baseline, baseline EDSS, brain MRI characteristics (baseline T2 lesions and CEL) and exposure to high-efficacy DMT. Natalizumab, rituximab and alemtuzumab were classified as high-efficacy DMTs, whereas teriflunomide, dimethyl fumarate, cladribine tablets, fingolimod and platform therapies were grouped as low/moderate efficacy DMT. Analyses and figures were created with RStudio (version 4.4.2). Core data examination and plotting were done using the packages readxl, dplyr, tidyr, tibble, ggplot2, ggrepel, stringr, pheatmap, VennDiagram, GridExtra and purrr. The packages MuMIn (model dredging), boot (CV), caret (model training), pROC (ROC curve and AUC analysis) and rms (survival modelling) were used for statistical modelling and performance evaluation. Differential expression analysis was performed using base stats functions together with the packages rstatix and ggpubr for hypothesis testing and significance annotation.

## Results

### CSF proteins were differentially expressed in RRMS and iPMS versus controls

We analysed the expression of 250 proteins (Supplementary Table 1) in CSF [controls (*n* = 44); RRMS (*n* = 49); iPMS (*n* = 32)] and plasma [controls (*n* = 49); RRMS (*n* = 20); iPMS (*n* = 20)] samples from 49 people with early RRMS, 33 people with inactive iPMS and 49 controls (Table 1). The control and RRMS groups did not differ in terms of age and sex. iPMS patients were older than both RRMS and controls (both *P* < 0.001). iPMS did not significantly differ from controls and RRMS in terms of sex (χ^2^, *P* = 0.102).

**Table 1:** Clinical, demographical and CSF parameters of study participants at baseline and follow-up

	Controls (*n* = 49)	RRMS (*n* = 49)	Stable RRMS (*n* = 35)	RRMS->SPMS (*n* = 14)	Non-PIRMA (*n* = 34)	PIRMA (*n* = 15)	iPMS (*n* = 33)
Baseline demographic and clinical parameters							
Age, years, median (IQR)	35.0 (21.0–58.5)	36.0 (28.0–46.0)	35.0 (28.0–45.0)	42.5 (31.8–48.5)	35.0 (28.0–43.0)	43.0 (33.0–47.0)	52.00 (36.00–57.50)
Sex, f, ***n*** (%)	32 (65.3)	35 (68.6)	24 (68.6)	9 (64.3)	23 (67.6)	10 (66.7)	15 (45.5)
Disease duration prior to sampling, m, median (IQR)		3.0 (1.0–6.0)	2.0 (0.0–5.0)	3.5 (2.7–9.2)	2.5 (0.0–6.0)	3.0 (2.0–6.0)	15 (6.0–22.0)
Baseline EDSS, median (IQR)		2.0 (1.5–3.25)	2.0 (1.5–2.5)	3.0 (1.8–3.5)	2.0 (1.5–2.5)	2.5 (1.5–3.5)	4.0 (3.0–6.0)
Baseline T2 lesions ≥ 10, ***n*** (%)		24 (49)	16 (45.7)	8 (57.1)	16 (47.1)	8 (53.3)	28 (84.8)
Baseline CEL (yes) ***n*** (%)		25 (51)	19 (54.3)	6 (42.9)	18 (52.9)	7 (46.7)	0 (0)
Follow-up clinical parameters							
Number of follow-up visits with assessment of EDSS, median (IQR)		12.0 (11.0–15.5)	13.0 (12.0–16.0)	12.0 (10.0–15.0)	12.5 (11.0–15.2)	12.0 (10.0–18.0)	9.0 (7.0–11.0)
Number of MRIs during follow-up, median (IQR)		12.0 (11.0–14.0)	13.0 (11.0–14.0)	11.5 (10.5–13.0)	12.5 (11.0–14.0)	12.0 (11.0–13.0)	8.0 (6.0–9.0)
EDSS at 6 years, median (IQR)		2.0 (0.0–3.0)	0.0 (0.0–2.0)	5.0 (3.5–6.5)	0.0 (0.0–2.0)	4.5 (2.5–6.0)	6.0 (4.0–6.5)
heDMT from start, ***n*** (%)		21 (42.9)	11 (31.4)	10 (71.4)	12 (35.3)	9 (60)	20 (60.6)
Dominant DMT, ***n*** (%)							
*No DMT*		0 (0)	0 (0)	0 (0)	0 (0)	0 (0)	8 (24.2)
*Interferons*		1 (2)	1 (2.9)	0 (0)	1 (2.9)	0 (0)	0 (0)
*Teriflunomide*		1 (2)	0 (0)	1 (7.1)	0 (0)	1 (6.7)	0 (0)
*Dimethyl fumarate*		14 (28.6)	12 (34.3)	2 (14.3)	11 (32.4)	3 (20)	0 (0)
*Cladribine tab*.		1 (2)	0 (0)	1 (7.1)	0 (0)	1 (6.7)	0 (0)
*Fingolimod*		3 (6.1)	3 (8.6)	0 (0)	3 (8.8)	0 (0)	0 (0)
*Rituximab*		9 (18.4)	6 (17.1)	3 (21.4)	6 (17.6)	3 (20)	25 (75.8)
*Natalizumab*		13 (26.5)	7 (20)	6 (42.9)	7 (20.6)	6 (40)	0 (0)
*Alemtuzumab*		7 (14.3)	6 (17.1)	1 (7.1)	6 (17.6)	1 (6.7)	0 (0)
Baseline CSF parameters							
WBC ≥ 5/μl, years/***n*** (range/μl)	0/49 (0–3)	24/25 (2–31)	16/19 (3–31)	8/6 (2–16)	15/19 (3–31)	8/7 (2–29)	4/29 (3–10)
IgG OCB ≥ 2, ***n*** (%)	0 (0)	47 (96)	33 (94.3)	14 (100)	32 (94.1)	15 (100)	33 (100)
Qalb, median (IQR)	3.6 (2.7–4.7)	4.7 (3.3–6.2)	4.6 (3.2–5.4)	6.2 (3.2–7.8)	4.7 (3.3–5.5)	4.8 (3.1–7.3)	5.2 (4.25–6.4)
IgG index, median (IQR)	0.52 (0.48–0.56)	0.81 (0.66–1.08)	0.81 (0.66–1.29)	0.77 (0.62–0.89)	0.79 (0.65–1.14)	0.81 (0.64–0.99)	0.76 (0.54–1.16)

CEL, contrast-enhancing lesions; CSF, cerebrospinal fluid; EDSS, expanded disability status scale; heDMT, highly effective disease-modifying therapy; IgG, immunoglobulin G; iPMS, inactive progressive multiple sclerosis; IQR, interquartile range; MRI, magnetic resonance imaging; OCB, oligoclonal bands; Qalb, albumin quotient; RRMS, relapsing-remitting multiple sclerosis; WBC, white blood cell.

**Figure 1:**
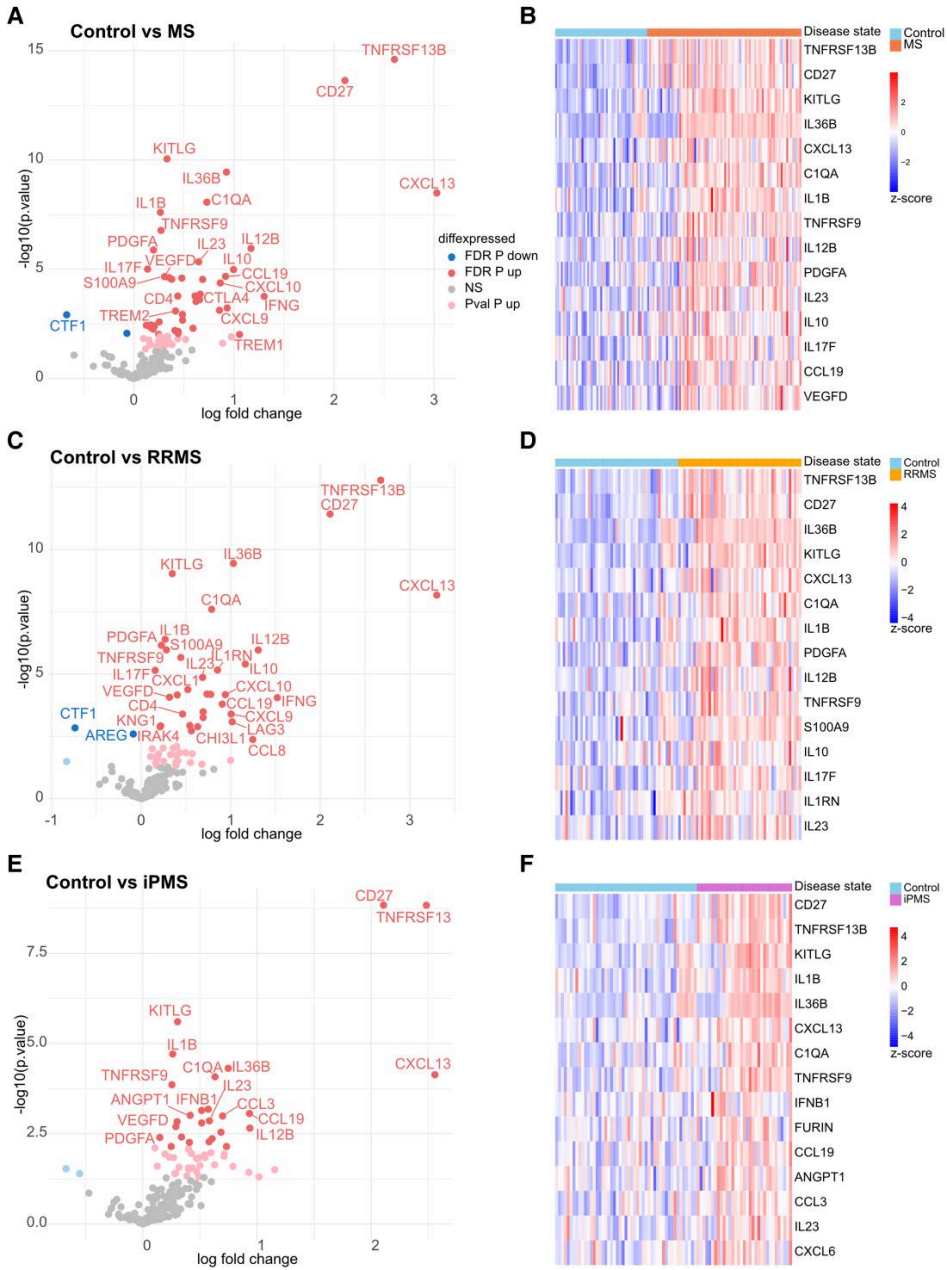
**Differential protein expression in CSF of patients with RRMS or iPMS compared with controls.** (**A, C, E**) Volcano plots depicting DEPs, (**B, D, F**) Heatmaps depicting the top 15 DEPs (ranked by FDR-adjusted *P*-value) in each comparison with heatmap colours representing row-scaled log_2_ protein expression values (*z*-score), indicating relative expression levels across samples. (**A, B**) MS compared with control, (**C, D**) in RRMS compared with Control and (**E, F**) in iPMS compared with Control. DEPs were identified by linear modelling with disease state as predictor and adjusted for age and sex and adjusted for multiple testing using the Benjamini–Hochberg method to control for FDR. Proteins with FDR-adjusted *P*-values < 0.05 were considered statistically significant. Proteins with unadjusted (raw) *P*-values < 0.05 but not significant after FDR correction are shown as nominally significant (*P*val-*P*) in lighter shade, grey are non-significant (NS). *n*_Control_ = 49, *n*_MS_ = 81, *n*_RRMS_ = 49, *n*_iPMS_ = 32.

**Figure 2:**
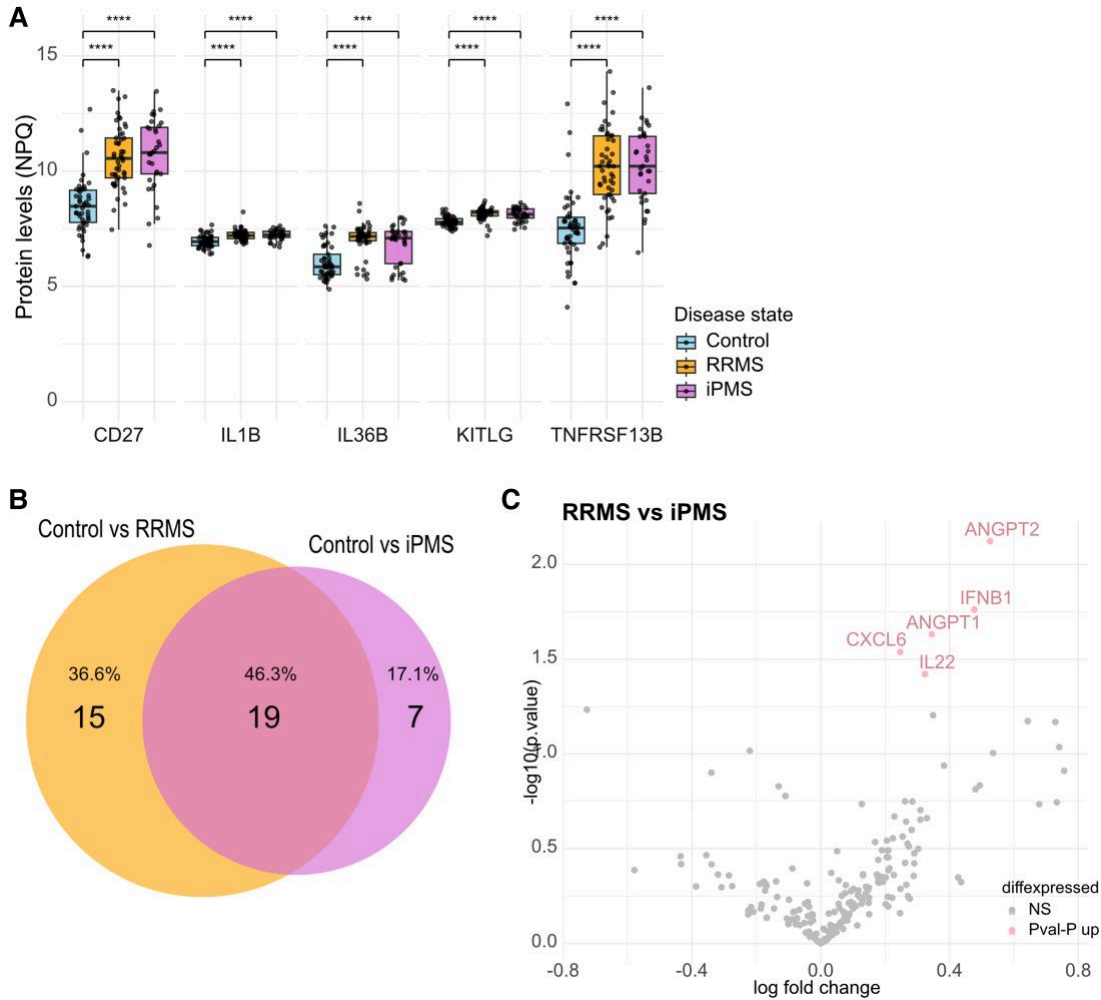
**Differentially expressed proteins in CSF of RRMS and iPMS patients.** (**A**) Boxplots for the protein levels (NPQ values) of the top five proteins differing in RRMS and iPMS compared with control, each dot represents one patient (experimental unit = patient), protein levels as pairwise comparison done with *t*-test. (**B**) Venn diagram showing shared and non-shared DEPs in RRMS and iPMS each compared with Control. (**C**) Volcano plot showing DEPs in iPMS compared with RRMS. DEPs were identified by linear modelling with disease state as predictor and adjusted for age and sex and adjusted for multiple testing using the Benjamini–Hochberg method to control for FDR. Proteins with FDR-adjusted *P*-values < 0.05 were considered statistically significant. Proteins with unadjusted (raw) *P*-values < 0.05 but not significant after FDR correction are shown as nominally significant (*P*val-*P*) in lighter shade, grey are non-significant (NS). *n*_Control_ = 49, *n*_MS_ = 81, *n*_RRMS_ = 49, *n*_iPMS_ = 32, ****P < 0.0001.

The 49 included controls consist of two control groups, healthy controls (H-Control) and symptomatic controls (S-Controls), that were combined as no significant differences could be observed (Supplemental Fig. 1A and B). Symptomatic controls consisted of people who underwent an investigation due to sensory symptoms (*n* = 18), functional motor disturbances (*n* = 11) and non-specific visual disturbances (*n* = 6). First, we compared the protein expression in CSF and plasma by merging the disease groups into a single MS group and comparing it to controls using ordinary least squares linear models. We found 46 proteins to be significantly upregulated in the CSF of MS patients compared with controls (top 10 DEPs: TNFRSF13B, CD27, KITLG, IL36B, CXCL13, C1QA, IL1B, TNFRSF9, IL12B and PDGFA) (Fig. 1A and B). No DEPs could be identified in plasma comparing MS and Controls (Supplemental Fig. 1C). Next, we split the disease groups into RRMS and iPMS and compared them, separately, with controls using ordinary least squares linear models. In RRMS, we found 34 proteins to be upregulated in the CSF (FDR *P* < 0.05) (top 10 DEPs: TNFRSF13B, CD27, IL36B, KITLG, CXCL13, C1QA, IL1B, PDGFA, IL12B and TNFRSF9) (Fig. 1C and D). No DEPs were found in plasma (Supplemental Fig. 1D). Similarly, in iPMS, we found 26 upregulated DEPs (top 10 DEPs: CD27, TNFRSF13, KITLG, IL1B, IL36B, CXCL13, C1QA, TNFRSF9, IFNB1 and FURIN) (Fig. 1E and F) and none in plasma (Supplemental Fig. 1E). Looking at the overall top five proteins (CD27, IL1B, IL36B, KITLG, TNFRSF13B), we can confirm significantly elevated protein levels in RRMS and iPMS patients compared with controls (Fig. 2A). RRMS and iPMS shared 19 upregulated DEPs, while 15 were only significantly upregulated in RRMS, and 7 were significantly upregulated in iPMS (Fig. 2B). After correction for FDR, no DEPs were found comparing RRMS and iPMS, neither in CSF (Fig. 2C) nor in plasma (Supplemental Fig. 1F).

While no plasma proteins passed FDR correction, plasma GFAP was nominally higher in iPMS versus controls (Supplemental Fig. 1G), and CSF GFAP was significantly elevated in iPMS compared with both RRMS and controls (Supplemental Fig. 1H), reflecting the expected astroglial activation in progressive MS.

### Top DEPs may assist in differentiating between RRMS and iPMS at baseline

**Figure 3:**
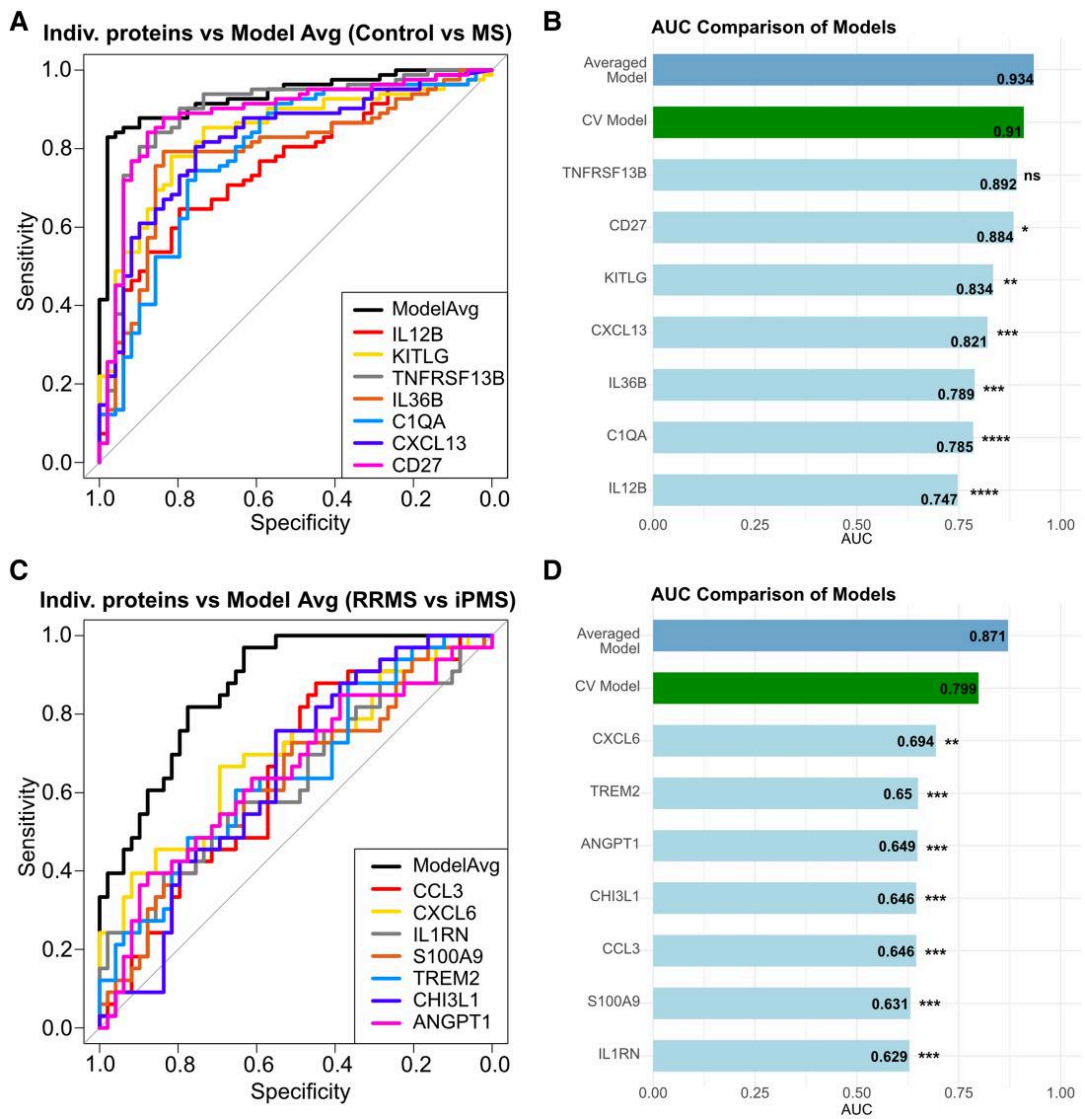
**Top DEPs can predict distinction between RRMS and iPMS.** (**A**) ROC curves for the averaged model distinguishing between Control and MS and for the individual proteins contributing to that model. (**B**) Bar plot visualizing the AUC of the averaged model, 10-fold cross-validated model and the individual proteins from **A**. DeLong’s test was performed to compare AUCs of individual protein models against the averaged model (*P*_TNFRSF13B_ = 0.076, *P*_CD27_ = 0.025, *P*_KITLG_ = 0.0027, *P*_CXCL13_ = 0.0006, *P*_IL36B_ = 0.0002, *P*_C1QA_ = 0.0001, *P*_IL12B_ = 4.74 × 10^−6^). (**C**) ROC curves of averaged model distinguishing between RRMS and PMS and of individual proteins contributing to that model. (**D**) Bar plot visualizing the AUCs of the average model, the cross-validated model and the individual proteins from **C**. DeLong’s test was performed to compare AUCs of individual protein models against averaged model (*P*_CCL3_ = 0.0002, *P*_CXCL6_ = 0.0039, *P*_IL1RN_ = 0.0004, *P*_S100A9_ = 0.00005, *P*_TREM2_ = 0.0004, *P*_CHI3L1_ = 0.0002, *P*_ANGPT1_ = 0.0009). *n*_Control_ = 49, *n*_MS_ = 81, *n*_RRMS_ = 49, *n*_iPMS_ = 32, *P < 0.05, **P < 0.01, ***P < 0.001, ****P < 0.0001.

We next wanted to assess, whether the identified DEPs can be used to distinguish the disease group classification. Performing model averaging, we identified a model consisting of IL12B, KITLG, TNFRSF13B, IL36B, C1QA, CXCL13 and CD27. This model discriminated between control and MS samples with high precision (ROC AUC = 0.934, 95% CI: 0.892–0.976, *P* < 0.001), outperforming any of the individual proteins (AUC = 0.747–0.892, all *P* < 0.05, *P*_TNFRSF13B_ = 0.076) (Fig. 3A and B). Following a 10-fold CV, the model retained strong performance (CV AUC = 0.91) (Fig. 3B).

Similarly, we applied model averaging and identified a model consisting of CCL3, CXCL6, IL1RN, S100A9, TREM2, CHI3L1 and ANGPT1 that distinguished between RRMS and iPMS groups with high precision (ROC AUC = 0.871, 95% CI: 0.799–0.944, *P* < 0.001), which, after 10-fold CV, presented an AUC of 0.799. Again, this model outperformed the predictive ability of any of the individual proteins (all individual proteins, *P* < 0.01 versus averaged model) (Fig. 3C and D).

### Low KITLG concentration at baseline is associated with higher risk of conversion to SPMS

**Figure 4:**
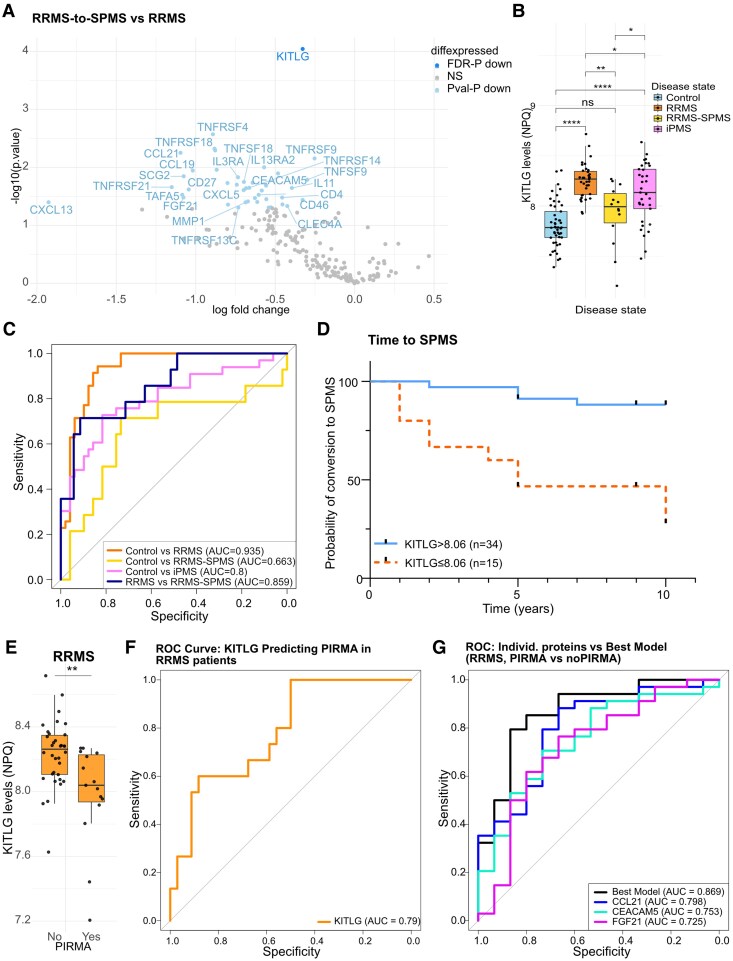
**KITLG-levels can distinguish between RRMS and RRMS-to-SPMS patients in CSF.** (**A**) Volcano plot depicting DEPs in CSF in RRMS-to-SPMS patients compared with RRMS patients. (**B**) Box plot of KITLG concentrations (NPQ) across all four patient groups, each dot represents one CSF sample (experimental unit = CSF sample). (**C**) ROC curve analysis for KITLG to distinguish Controls from MS subtypes, and RRMS-to-SPMS from RRMS. (**D**) Kaplan–Meier curve showing KITLG’s predictive value for conversion from RRMS to SPMS. (**E**) Kitlg-levels (NPQ) in RRMS patients separated for PIRMA events, each dot represents one CSF sample (experimental unit = CSF sample). (**F**) ROC curve showing the predictive ability of KITLG to distinguish between PIRMA and non-PIRMA. (**G**) ROC curve of the best-performing model and its individual proteins to predict PIRMA. *n*_Control_ = 49, *n*_iPMS_ = 32, *n*_RRMS_ = 35, *n*_RRMS-to-SPMS_ = 14, *n*_PIRMA_ = 15, *n*_non-PIRMA_ = 34, *P < 0.05, **P < 0.01, ***P < 0.001, ****P < 0.0001. DEPs are identified by linear modelling with disease state as predictor and adjusted for age, sex and treatment, and adjusted for multiple testing using the Benjamini–Hochberg method to control for FDR. Proteins with FDR-adjusted *P*-values < 0.05 were considered statistically significant. Proteins with unadjusted (raw) *P*-values < 0.05 but not significant after FDR correction are shown as nominally significant (*P*val-*P*) in lighter shade, grey are non-significant (NS).

Next, we analysed protein expression in CSF in RRMS patients who remained stable, compared with RRMS patients who later converted to SPMS. There were no significant differences between the groups in terms of age or sex. Only KITLG was found to be significantly downregulated in the CSF of patients who converted to SPMS (FDR *P* = 0.005) (Fig. 4A).

Hereafter, we opted to focus on CSF KITLG and its predictive ability in terms of conversion to SPMS and future PIRMA events. The cohort included 49 CSF samples from patients with early, untreated RRMS. Of these, 35 remained stable along the whole follow-up time [median 9 years, IQR (9–10)], whereas 14 converted to SPMS. In addition, 15 patients exhibited at least one sustained PIRMA event along the follow-up time. KITLG concentrations were highest in stable RRMS [median 8.27 IQR (8.11–8.35)] compared with controls [7.79 (7.68–7.97)], RRMS who converted to SPMS [7.99 (7.76–8.17)] and iPMS [8.13 (7.91–8.38)] (Fig. 4B). A ROC curve analysis demonstrated an AUC of 0.935 (95% CI: 0.883–0.987, *P* < 0.001) for Controls versus RRMS, and an AUC of 0.80 (95% CI: 0.679–0.903, *P* < 0.001) for Controls versus iPMS, suggesting an overall good predictive role for KITLG in differentiating between controls and RRMS or iPMS samples (Fig. 4C). However, the ROC curve for Control versus RRMS-to-SPMS only achieved an AUC of 0.66 (95% CI: 0.472–0.854, *P* = 0.0936) (Fig. 4C). Of specific interest is also, that a ROC curve for KITLG for RRMS versus RRMS-to-SPMS presented an AUC of 0.859 (95% CI: 0.744–0.974, *P* < 0.001), demonstrating the protein’s relevance in distinguishing between RRMS and RRMS-to-SPMS samples (Fig. 4C). In contrast, RRMS-to-SPMS versus iPMS presented an AUC of 0.690, while the AUC of a ROC curve for RRMS versus iPMS was 0.603 (Supplemental Fig. 2A). We used the Youden index to calculate the most discriminative cut-off to differentiate between stable RRMS and conversion to SPMS to be >8.06 NPQ. Next, to investigate the association of KITLG with lower risk of SPMS conversion, we built a multivariable Cox proportional hazards model adjusting for age, sex, disease duration, baseline T2 lesions and CELs, as well as treatment category. Testing KITLG as a continuous variable, increasing concentrations were associated with a protective effect (adjusted hazard ratio [aHR] 0.02, 95% CI: 0.001–0.52, *P* = 0.019). Testing KITLG as a categorical variable based on the cut-off >8.06, KITLG ≤ 8.06 was associated with higher SPMS conversion hazard (aHR 5.01, 95% CI: 1.26–19.87, *P* = 0.022) (Fig. 4D and Supplementary Table 1).

### Low baseline KITLG concentration is associated with PIRMA

We further dichotomized the RRMS cohort into PIRMA/non-PIRMA. Of the 49 RRMS patients with available CSF samples, 15 (30.6%) exhibited a sustained PIRMA event along the follow-up. Only two patients who exhibited a sustained PIRMA event did not go on to develop SPMS during the study follow-up. RRMS patients with a PIRMA event had lower CSF KITLG concentrations at baseline (8.04 IQR 7.92–8.24) compared with the non-PIRMA group (8.26 IQR 8.10–8.35) (*P* = 0.002) (Fig. 4E). There were no differences between the groups in terms of age or sex. The ROC curve using KITLG as predictor for PIRMA had an AUC of 0.786 (95% CI: 0.653–0.920, *P* < 0.001) (Fig. 4F). Again, testing KITLG as a continuous variable revealed a protective effect with each increasing NPQ unit (aHR 0.066, 95% CI: 0.01–0.46, *P* = 0.006) (Fig. 4D). However, logistic regression analysis based on proteins retaining a significant *P*-value and logFC > 0.8 (data not shown) identified a multivariable model consisting of CSF CCL21, CEACAM5 and FGF21 as the best-performing combination for distinguishing between PIRMA and non-PIRMA RRMS patients (AUC = 0.869, 95% CI: 0.754–0.984, *P* < 0.001). This model outperformed the predictive ability of each individual protein (Fig. 4G).

### Plasma concentrations of two proteins are associated with conversion to SPMS

**Figure 5:**
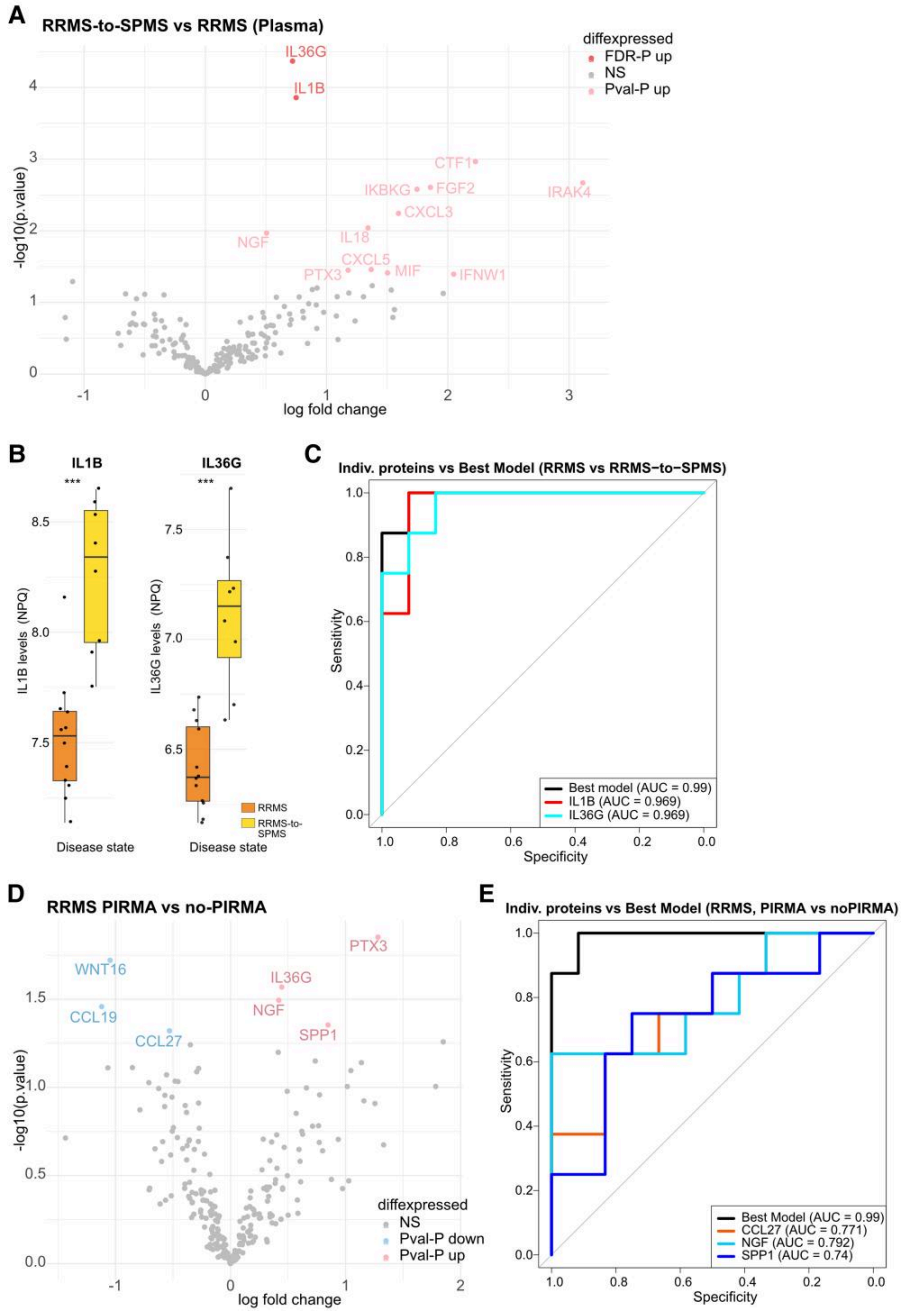
**Six proteins differentiate between RRMS and RRMS converting to SPMS in plasma.** (**A**) Volcano Plot depicting DEPs in plasma of RRMS-to-SPMS patients compared with RRMS patients. (**B**) Boxplots of the protein levels (NPQ) of the two identified DEPs after FDR correction, each dot represents one plasma sample (experimental unit = plasma sample). (**C**) ROC curve of best predictive model and its individual proteins to distinguish between RRMS-to-SPMS and RRMS in plasma. (**D**) Volcano plot depicting DEPs in plasma of RRMS patients with and without PIRMA. (**E**) ROC curve of the best predictive model and its individual proteins to distinguish between PIRMA and no-PIRMA in RRMS patients. *n*_RRMS_ = 12, *n*_RRMS-to-SPMS_ = 8, *n*_PIRMA_ = 8, *n*_non-PIRMA_ = 12, *P < 0.05, **P < 0.01, ***P < 0.001, ****P < 0.0001. DEPs are identified by linear modelling with disease state as predictor and adjusted for age, sex and treatment and adjusted for multiple testing using the Benjamini–Hochberg method to control for FDR. Proteins with FDR-adjusted *P*-values < 0.05 were considered statistically significant. Proteins with unadjusted (raw) *P*-values < 0.05 but not significant after FDR correction are shown as nominally significant (*P*val-*P*) in lighter shade, grey are non-significant (NS).

Although plasma did not distinguish diagnostic groups cross-sectionally, we reasoned that systemic inflammatory changes may precede clinical deterioration, and therefore returned to plasma to evaluate its prognostic utility in identifying RRMS patients at risk of progression. Therefore, we further analysed protein levels in the plasma of patients with stable RRMS and patients that converted to SPMS. Interestingly, two proteins were found to be significantly upregulated in plasma samples from RRMS patients who converted to SPMS (FDR *P* < 0.05) compared with stable RRMS (Fig. 5A). Specifically, IL1B and IL36G were significantly upregulated in the plasma of RRMS patients who convert to SPMS compared with stable RRMS (Fig. 5B). IL1B was markedly higher in RRMS patients who converted to SPMS [8.34 (7.95–8.55)] compared with stable RRMS [7.53 (7.33–7.64)] (FDR *P* = 0.0047). Similarly, IL36G was elevated in converters [7.15 (6.92–7.27)] versus non-converters [6.37 (6.26–6.60)] (FDR *P* = 0.004). To evaluate their predictive potential, we performed a model selection analysis, which identified a two-protein logistic regression model including IL1B and IL36G as the best-performing combination (AUC = 0.990, 95% CI: 0.961–1.000, *P* < 0.001), slightly outperforming the individual proteins (IL1B: AUC = 0.969, IL36G: AUC = 0.969; Fig. 5C). Next, we assessed whether any of these proteins are also suitable to distinguish between PIRMA and non-PIRMA plasma samples. In contrast to the conversion of RRMS to SPMS, no proteins remained significant after FDR correction. However, seven proteins were significantly altered after raw *P*-value, including IL36G [7.04 (6.96–7.22) versus 6.40 (6.32–6.64), *P* = 0.0141] which were upregulated in PIRMA compared to non-PIRMA (Fig. 5D). From these seven altered proteins, we could identify a prediction model (Best Model) for PIRMA consisting of CCL27, NGF and SPP1 outperforming any of the individual proteins alone (AUC = 0.990, 95% CI: 0.961–1.000, *P* < 0.001) (Fig. 5E).

## Discussion

Our exploratory analysis demonstrates that the CSF sub-proteome in patients with RRMS onset is largely dominated by markers that reflect adaptive immune responses (e.g. TNFRSF13B, CD27, CXCL13 and TNFRSF9), and while these proteins remain upregulated in iPMS compared with controls, there is a noticeable shift towards innate immune activation in iPMS (e.g. IL1B, C1QA, IL36B, IFNB1, CCL3 and CXCL6). These results are largely confirmatory and strengthen our confidence that the NULISA inflammatory panel provides an accurate representation of the CSF sub-proteome within the MS continuum.

Our findings align with and expand upon recent proteomic studies investigating the role of inflammation in MS progression. Notably, recent studies identified a combination of CSF proteins, including CXCL13, CHI3L1 and TNFRSF13B, that could predict long-term disability worsening in MS patients with high accuracy.^18,19^ Our confirmation of these markers supports their role in MS pathogenesis and highlights the robustness of proteomic-based biomarker discovery. Moreover, both our study and another recent proteomic analysis^20^ found only minimal proteomic differences between RRMS and iPMS, reinforcing the notion that disease progression is likely driven by smouldering inflammation rather than distinct mechanistic shifts during transitions between phenotypes. In our cohort, plasma markers had modest cross-sectional discriminatory power, but several individual proteins and multivariable combinations nevertheless showed encouraging prognostic potential, particularly for identifying patients at risk of progression. These findings contrast with a large-scale plasma proteomic study, where more than 70 plasma proteins were significantly altered in MS compared with controls, including established markers such as GFAP and NfL.^21^ The limited plasma findings in our study are likely influenced in part by the relatively small plasma sample size, which reduces power to detect subtle but biologically meaningful group differences. The absence of FDR-significant GFAP differences in plasma, despite a nominal elevation in iPMS, is likely explained by the relatively small plasma sample size and the comparatively short disease duration in our cohort. Both factors reduce the expected dynamic range of plasma GFAP, which typically increases most robustly in larger cohorts and in patients with longer-standing or more advanced neurodegeneration.

The model distinguishing MS from controls, composed of IL12B, KITLG, TNFRSF13B, IL36B, C1QA, CXCL13 and CD27, achieved an AUC of 0.934 and remained strong after CV (AUC = 0.916). These proteins are all implicated in immune regulation and CNS inflammation. Notably, TNFRSF13B, CXCL13 and CD27 have been repeatedly associated with MS activity in previous CSF studies.^22-24^ IL12B and TNFRSF13B are key mediators in Th1 and B-cell responses, respectively, both of which are critical in MS pathogenesis. C1QA, a complement component, may reflect broader innate immune activation. CSF C1q levels have previously been shown to be elevated in RRMS patients compared with healthy controls.^25,26^ Interestingly, higher CSF C1q levels were found to be associated with lower disability progression in PPMS patients,^27^ potentially due to its neuroprotective and anti-inflammatory properties.^28^

Similarly, our RRMS versus iPMS model, composed of CCL3, CXCL6, IL1RN, S100A9, TREM2, CHI3L1 and ANGPT1, also showed a strong predictive performance (AUC = 0.871; CV AUC = 0.823). These markers collectively point to heightened activation of microglia and monocyte–macrophage pathways (CCL3, S100A9 and TREM2), increased neutrophil-associated chemokine signalling (CXCL6), counter-regulatory cytokine responses within the IL-1 axis (IL1RN), astroglial activation and tissue remodelling (CHI3L1) and altered angiogenic/tissue integrity pathways (ANGPT1). Taken together, this pattern supports the concept that progression in MS is accompanied by a shift from adaptive immune activity towards compartmentalized innate immune and glial-mediated mechanisms within the CNS.

Importantly, both multivariate models outperformed the predictive ability of individual proteins. This highlights the added value of combinatorial biomarker approaches in capturing the complex immunopathology of MS, in contrast to reliance on single biomarkers, which may have limited sensitivity or specificity. While promising, our models require validation in independent cohorts and clinical settings to assess generalizability and potential utility in guiding therapeutic decisions.

We identified IL1B and IL36G as plasma markers associated with conversion to SPMS. This suggests a more significant role for systemic inflammation in MS progression, warranting further investigation. IL1B was furthermore increased in the CSF of RRMS and iPMS patients compared with controls. IL1B is a key pro-inflammatory cytokine that plays a crucial role in innate immune activation and has been implicated in neuroinflammation and neurodegeneration. IL1B release is tightly linked to activation of the NLRP3 inflammasome, a multiprotein complex that mediates caspase-1 activation and the subsequent cleavage of IL1B and IL18. The NLRP3 inflammasome is activated in response to cellular stress and damage-associated molecular patterns (DAMPs),^29^ and has been increasingly recognized as a key driver of chronic inflammation in progressive MS.^30^ Activated microglia, a hallmark of progressive MS pathology, have been shown to exhibit heightened NLRP3 inflammasome activity, leading to sustained IL1B production, oxidative stress and neurodegenerative cascades.^31^ Elevated IL1B levels in plasma may reflect systemic immune activation, which could contribute to neuroinflammation within the CNS and promote a progressive disease course.

In the context of our findings, the marked upregulation of IL1B in RRMS patients who convert to SPMS may indicate an early inflammatory signature that predisposes individuals to a progressive disease trajectory. This aligns with emerging evidence that smouldering inflammation, characterized by persistent microglial activation and low-grade inflammatory responses, underlies disease progression in MS. Moreover, IL1B has been shown to impair remyelination by affecting oligodendrocyte precursor cell (OPC) differentiation, further linking its role to MS progression.^32^

Given these observations, plasma IL1B could serve as a potential biomarker for identifying RRMS patients at risk of transitioning to SPMS. Targeting IL1B or upstream NLRP3 inflammasome activation may represent a novel therapeutic approach to mitigate neuroinflammation and slow disease progression in MS. Future studies should focus on validating IL1B as a prognostic biomarker and exploring inflammasome inhibitors as potential DMTs for progressive MS.

Our analysis also reveals important novel findings. In both RRMS and iPMS, CSF KITLG emerged as one of the top five upregulated proteins. Intriguingly, KITLG appeared to be particularly upregulated in patients with stable RRMS compared with those who later develop SPMS. KITLG, also known as stem cell factor, is a cytokine that plays a critical role in haematopoiesis, melanogenesis and gametogenesis by acting as a ligand for the c-kit receptor (CD117).^33,34^ The KITLG/c-kit axis is also involved in various physiological and pathological processes, such as cell proliferation, survival and migration.^35-37^ Direct evidence on KITLG secretion by CNS cells and its measurement in CSF is limited, although one study has previously demonstrated elevated KITLG concentrations in the CSF of MS patients.^38^ KITLG has been demonstrated to cross the blood–brain barrier and thereafter influences neurogenesis and neuroprotection.^39^ Previous studies have shown that KITLG is predominantly localized to neuronal populations in the normal brain. However, both neurons and glial cells, including astrocytes and microglia, express its transmembrane receptor with tyrosine kinase activity, encoded by the proto-oncogene *c-kit*.^40^ This distribution suggests that KITLG/c-kit signalling mediates interactions between neurons, as well as between neurons and glial cells.^41-43^ KITLG has been shown to increase microglial expression of mRNAs encoding neurotrophic factors such as nerve growth factor (*NGF*), brain-derived neurotrophic factor (*BDNF*) and ciliary neurotrophic factor (*CNTF*). Concurrently, KITLG downregulates microglial expression of pro-inflammatory cytokines, including tumour necrosis factor-α (TNF-α) and IL1B.^44^ These findings indicate that KITLG/*c-kit* signalling enhances the neurotrophic support potential of microglia while attenuating their pro-inflammatory effects, mechanisms that may play a role in the pathophysiology of inflammatory CNS disorders, including progressive MS.

In our study, we observed significantly higher baseline concentrations of KITLG in individuals with stable RRMS compared with those who later converted to SPMS. The AUC of 0.85 suggests that KITLG is a potential predictive biomarker for distinguishing at baseline stable RRMS from those individuals at higher risk of progression. Furthermore, the survival analysis (log-rank *P* < 0.0001) comparing groups with KITLG levels >8.06 NPQ and <8.06 NPQ underlines the potential ability of KITLG in identifying patients with a favourable versus unfavourable disease trajectory. KITLG is a key factor in supporting the survival and proliferation of OPCs, which are critical for remyelination. Higher levels of KITLG may reflect an enhanced capacity for neuroprotection and repair in stable RRMS patients, allowing better recovery from relapses and preservation of neuronal function. In contrast, individuals who later convert to SPMS may have lower KITLG levels, reflecting a diminished capacity for repair and resilience to cumulative CNS damage. Furthermore, lower KITLG levels in patients prone to progression might reflect impaired immune regulation, allowing chronic, low-grade inflammation to drive the transition to SPMS. KITLG is essential for mast cell survival and function.^45,46^ In stable RRMS, higher KITLG levels might promote beneficial mast cell activity, such as releasing neurotrophic factors that aid in tissue repair. This protective effect could help limit long-term damage. In patients with lower KITLG levels, reduced mast cell activity might contribute to chronic neuroinflammation driven by activated microglia. Finally, KITLG plays a role in maintaining endothelial cell function and BBB integrity.^47,48^ Higher KITLG levels in stable RRMS could indicate a more intact BBB, reducing the infiltration of peripheral immune cells into the CNS and protecting against sustained inflammation.

Several recent studies have demonstrated that intrathecal indices, which combine CSF and serum concentrations while correcting for blood–CSF barrier permeability, can markedly improve the prognostic value of inflammatory biomarkers in MS. The best-established example is the CXCL13 index, which has consistently outperformed CSF CXCL13 alone for predicting future disease activity. In one study, the CXCL13 index showed superior discrimination of upcoming clinical or MRI activity compared with OCBs, CSF CXCL13 concentrations and CSF neurofilament light (NfL),^49^ highlighting the value of quantifying intrathecal chemokine production rather than absolute CSF levels. More recently, another study demonstrated that the CXCL13 index predicted the success or failure of moderate-efficacy DMTs in a real-world cohort,^50^ further underscoring its clinical utility. These observations suggest that intrathecal indices may enhance the performance of several candidate biomarkers identified in our study, particularly for chemokine- and cytokine-related pathways where serum levels and barrier permeability strongly influence CSF concentrations. Future work incorporating intrathecal indices for proteins such as KITLG, CCL21 and PTX3 may therefore provide a more accurate reflection of compartmentalized CNS inflammation and improve prediction of both inflammatory and progression-related outcomes.

A major strength of this study is the application of NULISA, a highly sensitive and scalable proteomic platform, to analyse CSF and plasma in a well-characterized MS cohort with long-term clinical follow-up. The identification of robust multivariate biomarker models with strong cross-validated performance enhances the translational relevance of our findings. Furthermore, the integration of molecular data with clinical endpoints such as PIRMA and SPMS conversion provides insight into disease mechanisms and prognostication.

Nevertheless, several limitations must be acknowledged. First, the sample size, particularly for plasma analyses and subgroup comparisons, was modest and may limit statistical power. Second, while we adjusted for known confounders, residual confounding cannot be excluded. Third, external validation in independent cohorts is needed to confirm the generalizability of the biomarker models. In addition, the targeted nature of the NULISA inflammatory panel necessarily limits the breadth of proteins measured compared with untargeted or high-coverage proteomic platforms. Finally, although KITLG and IL1B are mechanistically plausible biomarkers, direct experimental validation of their roles in MS pathophysiology remains necessary.

In summary, our study reveals CSF proteomic profiles that differentiate RRMS and iPMS patients from controls and suggests that smouldering innate immune activation is a core feature of progressive MS. KITLG emerged as a potential CSF biomarker associated with protection against both conversion to SPMS and future PIRMA events, while IL1B, IKBKG and IL36G in plasma may serve as possible early indicators of progression risk. These findings highlight the potential utility of multiplex proteomics, such as NULISA, in uncovering clinically relevant molecular pathways in MS. While our multivariate models demonstrate strong predictive performance, validation in independent cohorts and integration with imaging and clinical data are essential steps towards clinical translation. Ultimately, the identification of molecular markers of smouldering MS may help refine patient stratification, inform treatment decisions and guide the development of targeted neuroprotective therapies.

## Supplementary Material

fcag142_Supplementary_Data

## Data Availability

The data that support the findings of this study are available from the corresponding author, upon reasonable request.
