# Supplementary material for: A novel multiplexed biomarker panel reveals key molecular pathways in progressive multiple sclerosis
Source: Brain Commun. 2026 Apr 17;8(3):fcag142. doi: 10.1093/braincomms/fcag142 (PMC13160671; doi:10.1093/braincomms/fcag142)
Supplement: fcag142_Supplementary_Data [file fcag142_supplementary_data.pdf]

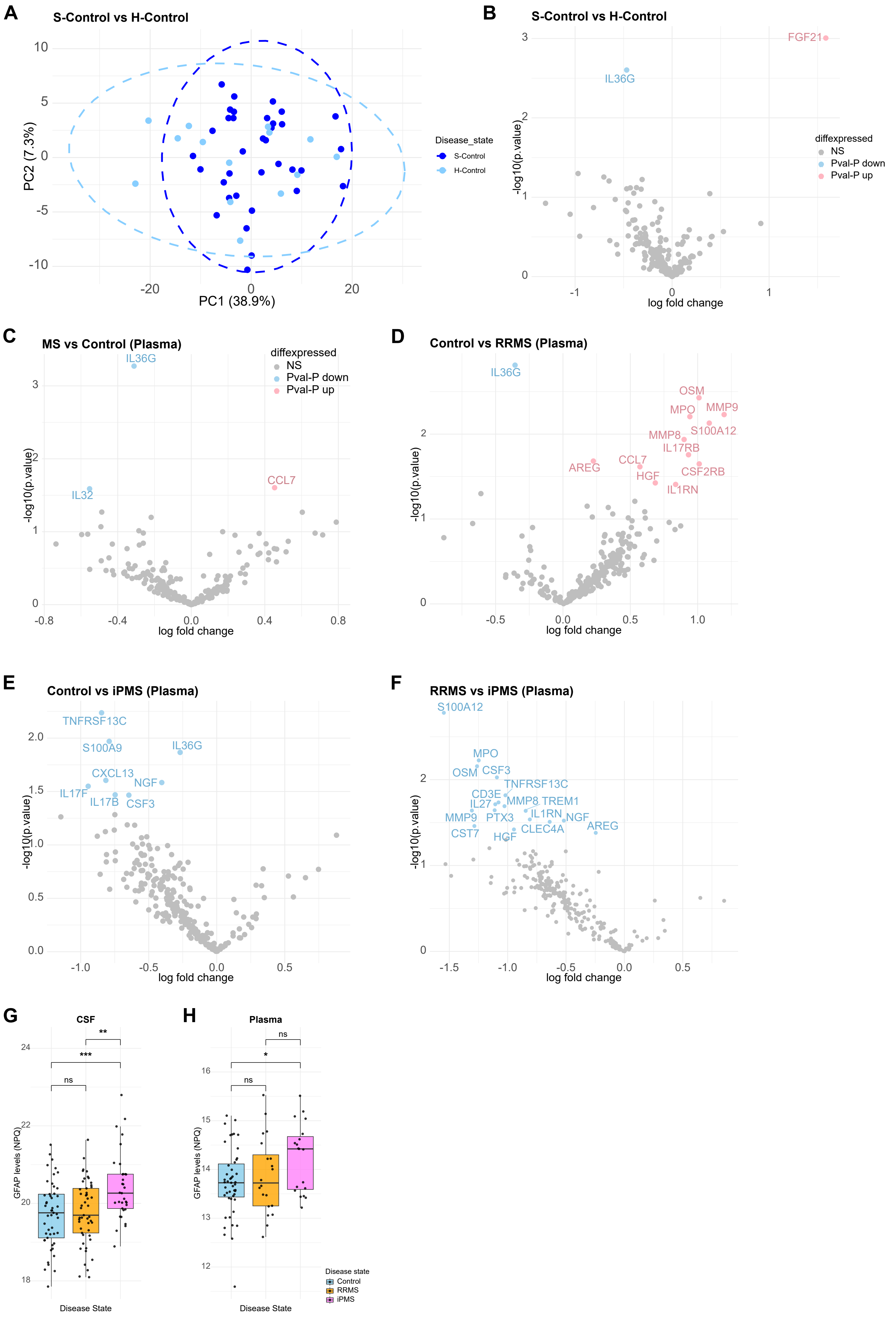
A
S-Control vs H-Control
PC1 (38.9%)
PC2 (7.3%)
Disease_state
S-Control
H-Control
B
S-Control vs H-Control
FGF21
IL36G
-log10(p.value)
log fold change
diffexpressed
NS
Pval-P down
Pval-P up
C
MS vs Control (Plasma)
IL36G
IL32
CCL7
D
Control vs RRMS (Plasma)
IL36G
OSM
MPO
MMP9
S100A12
MMP8
IL17RB
AREG
CCL7
HGF
CSF2RB
IL1RN
E
Control vs iPMS (Plasma)
TNFRSF13C
S100A9
IL36G
CXCL13
NGF
IL17F
IL17B
CSF3
F
RRMS vs iPMS (Plasma)
S100A12
MPO
OSM
CSF3
TNFRSF13C
CD3E
IL27
MMP8
TREM1
MMP9
PTX3
IL1RN
NGF
AREG
CST7
HGF
CLEC4A
G
CSF
GFAP levels (NPQ)
Disease State
H
Plasma
Disease state
Control
RRMS
iPMS

Supplemental Figure 1: Analysis of plasma to identify DEPs across various patient groups. (A) Principal component analysis showing that healthy (H) controls (n = 14) and symptomatic (S) controls (n = 35) do not form separate clusters; each datapoint represents one plasma sample (experimental unit = patient). (B) Volcano plot for DEPs in CSF of H-Controls compared with S-Controls (C) Volcano plot to identify DEPs in plasma of patients with MS compared with controls (D) Volcano plot for DEPs in RRMS patients compared with controls and (E) in PMS patients compared with (F) Volcano plot analyzing DEPs between RRMS and PMS patients (G) Boxplot for GFAP levels (NPQ) in CSF, (H) Boxplot for GFAP levels (NPQ) in Plasma, each data point represents one CSF (G) or plasma (H) sample (experimental unit = CSF/plasma sample). DEPs are identified by linear modelling with disease state as predictor, adjusted for age and sex, and adjusted for multiple testing using the Benjamini-Hochberg method to control for false-discovery rate (FDR), comparison between two groups (G-H): t-test. $n_{Control\_Plasma}$=47, n_{MS_Plasma}=40, n_{RRMS_Plasma}=20, n_{iPMS_Plasma}=20, $n_{Control}$=49, n_{RRMS}=49, n_{iPMS}=32, * $p<0.05$, ** $p<0.01$, *** $p<0.001$.

A

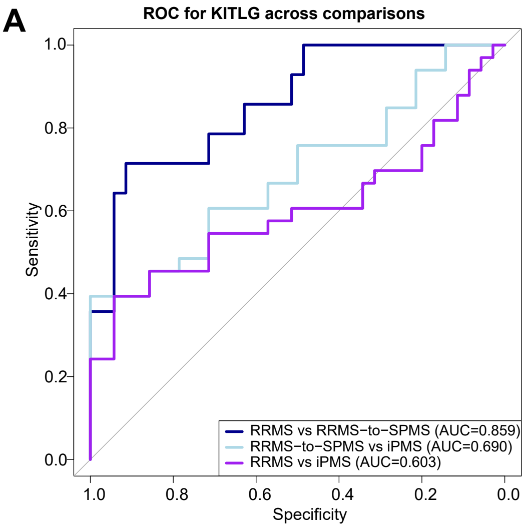

ROC for KITLG across comparisons
Sensitivity
Specificity
1.0
0.8
0.6
0.4
0.2
0.0
RRMS vs RRMS−to−SPMS (AUC=0.859)
RRMS−to−SPMS vs iPMS (AUC=0.690)
RRMS vs iPMS (AUC=0.603)

Supplemental Figure 2: KIT ligand distinguishes multiple sclerosis subgroups. (A) ROC curve showing the ability of KIT ligand levels in cerebrospinal fluid to discriminate between relapsing–remitting multiple sclerosis, relapsing–remitting multiple sclerosis converting to secondary progressive multiple sclerosis, and inactive progressive multiple sclerosis. n_{iPMS}=32, n_{RRMS}=35, $n_{RRMS\text{-}to\text{-}SPMS}$ = 14.

Supplemental Table 1: Inflammatory protein panel analyzed with NULISA. The table lists all proteins included in the inflammatory panel, together with their measured concentration ranges (interquartile ranges (Q1-Q3) in CSF and plasma samples from all patients, separated for Controls, RRMS, and iPMS patients. $n_{Control}$=49, n_{RRMS}=49, n_{iPMS}=32, $n_{Control\_Plasma}$=47, n_{MS_Plasma}=40, n_{RRMS_Plasma}=20, n_{iPMS_Plasma}=20.

Supplemental Table 1

Protein	CSF			PLASMA		
	Control (Q1Q3)	RRMS (Q1Q3)	iPMS (Q1Q3)	Control (Q1Q3)	RRMS (Q1Q3)	iPMS (Q1Q3)
**AGER**	5.32 - 6.82	5.53 - 6.78	5.48 - 6.45	13.49 - 14.07	13.68 - 14.41	13.34 - 14.11
**AGRP**	11.91 - 12.70	11.76 - 12.75	11.94 - 12.91	13.30 - 13.73	13.35 - 14.15	12.87 - 13.62
**ANGPT1**	7.33 - 8.00	7.48 - 8.14	7.81 - 8.38	11.87 - 13.19	11.96 - 12.99	12.09 - 13.02
**ANGPT2**	9.10 - 9.87	8.99 - 10.04	9.28 - 10.21	12.58 - 13.35	12.46 - 13.37	12.35 - 13.15
**ANXA1**	7.91 - 8.38	8.03 - 8.33	8.03 - 8.31	9.37 - 11.48	9.82 - 11.48	8.77 - 11.33
**AREG**	9.72 - 9.89	9.62 - 9.82	9.71 - 9.83	9.82 - 10.20	10.03 - 10.61	9.88 - 10.20
**BDNF**	0.00 - 0.00	0.00 - 0.00	0.00 - 0.00	12.83 - 14.89	12.53 - 15.14	12.96 - 14.63
**BMP7**	12.73 - 13.52	12.56 - 13.37	12.74 - 13.51	12.06 - 12.67	11.92 - 12.39	11.97 - 12.61
**BST2**	5.75 - 6.97	5.87 - 7.32	6.08 - 7.47	10.31 - 11.11	10.46 - 11.52	10.24 - 11.79
**C1QA**	8.19 - 9.00	9.13 - 9.81	8.75 - 9.73	12.49 - 13.03	12.39 - 13.50	12.36 - 13.07
**CALCA**	15.56 - 16.68	15.74 - 16.88	15.90 - 17.10	12.88 - 13.73	13.10 - 13.70	13.16 - 14.19
**CCL1**	11.36 - 12.42	11.40 - 12.61	11.66 - 12.50	12.78 - 13.39	12.84 - 13.68	12.22 - 13.20
**CCL11**	9.01 - 9.98	9.25 - 9.94	9.28 - 10.35	12.50 - 13.38	12.46 - 13.54	13.01 - 13.94
**CCL13**	4.30 - 6.28	4.43 - 6.51	4.32 - 6.40	12.59 - 13.49	12.39 - 13.41	12.91 - 13.57
**CCL14**	6.23 - 8.68	6.16 - 8.53	6.50 - 9.16	12.56 - 13.01	12.76 - 13.18	12.39 - 13.00
**CCL15**	0.00 - 4.67	0.00 - 4.91	0.00 - 5.19	12.22 - 13.45	12.42 - 13.91	12.33 - 13.27
**CCL16**	2.06 - 5.14	2.59 - 5.31	1.67 - 5.40	11.20 - 11.93	11.38 - 12.07	11.14 - 11.95
**CCL17**	5.15 - 7.04	5.23 - 7.31	6.15 - 7.14	12.26 - 14.02	12.08 - 13.92	12.20 - 13.49
**CCL19**	10.34 - 11.85	11.25 - 12.80	11.12 - 12.81	12.25 - 12.79	12.12 - 13.19	12.39 - 13.49
**CCL2**	12.96 - 14.33	12.66 - 14.06	13.27 - 14.57	12.02 - 12.94	12.01 - 12.90	12.33 - 12.98
**CCL20**	9.70 - 10.20	9.80 - 10.42	9.69 - 10.32	11.41 - 12.22	11.27 - 12.56	11.26 - 12.09
**CCL21**	6.60 - 7.67	6.45 - 7.80	6.67 - 7.81	13.21 - 13.72	13.19 - 13.55	13.02 - 13.94
**CCL22**	0.00 - 4.29	0.00 - 6.88	0.00 - 6.55	12.82 - 13.69	12.40 - 13.63	12.09 - 13.61
**CCL23**	5.63 - 7.37	5.75 - 7.30	5.21 - 8.03	12.87 - 13.75	13.19 - 13.60	12.75 - 13.49
**CCL24**	0.00 - 6.56	0.00 - 6.04	0.00 - 6.31	12.87 - 14.96	12.93 - 15.08	13.32 - 15.66
**CCL25**	0.00 - 4.79	0.00 - 4.28	0.00 - 5.35	12.13 - 12.97	12.20 - 13.05	12.03 - 13.60
**CCL26**	7.97 - 9.25	8.05 - 9.20	8.38 - 9.41	11.20 - 12.06	11.21 - 11.97	11.13 - 12.20
**CCL27**	4.00 - 6.05	4.17 - 5.67	4.29 - 6.38	13.25 - 14.02	13.45 - 13.98	13.29 - 14.10
**CCL28**	0.00 - 0.00	0.00 - 0.00	0.00 - 0.00	13.33 - 14.82	13.32 - 14.86	13.01 - 14.41
**CCL3**	9.40 - 10.82	10.06 - 11.14	10.45 - 11.55	11.69 - 12.44	11.70 - 12.67	11.73 - 12.62
**CCL4**	8.31 - 9.20	8.51 - 9.79	8.66 - 9.56	11.13 - 12.23	11.38 - 12.17	11.20 - 11.87
**CCL5**	0.00 - 0.00	0.00 - 0.00	0.00 - 0.00	12.38 - 15.33	11.94 - 15.10	12.23 - 15.21
**CCL7**	8.26 - 9.04	8.27 - 9.15	8.57 - 9.24	10.40 - 11.16	10.68 - 12.13	10.64 - 11.79
**CCL8**	7.13 - 9.28	7.98 - 9.66	8.18 - 9.53	11.69 - 13.19	11.55 - 13.29	12.35 - 13.21
**CD200**	16.82 - 18.00	15.89 - 17.85	16.18 - 17.81	12.91 - 14.15	12.99 - 14.23	4.98 - 13.59
**CD200R1**	0.00 - 7.10	0.00 - 7.11	5.11 - 6.97	12.86 - 13.33	13.03 - 13.75	12.85 - 13.49
**CD27**	7.78 - 9.18	9.71 - 11.44	9.88 - 11.90	12.77 - 13.25	12.78 - 13.13	12.75 - 13.26
**CD274**	11.53 - 12.46	11.17 - 12.50	11.55 - 12.59	13.09 - 13.47	13.04 - 13.60	13.03 - 13.40

**CD276**	8.13 - 9.18	8.00 - 9.09	8.25 - 9.15	13.55 - 13.98	13.56 - 14.01	13.37 - 13.95
**CD3E**	4.40 - 6.30	4.84 - 7.02	4.26 - 6.60	10.65 - 11.89	10.84 - 12.69	10.24 - 11.57
**CD4**	9.07 - 9.91	9.46 - 10.28	9.46 - 10.32	13.08 - 13.47	13.24 - 13.68	12.77 - 13.48
**CD40**	10.55 - 11.35	10.46 - 11.71	10.61 - 12.04	12.76 - 13.25	12.84 - 13.30	12.83 - 13.43
**CD40LG**	3.88 - 5.73	4.07 - 6.34	4.20 - 5.52	11.58 - 14.24	11.82 - 13.99	11.85 - 13.49
**CD46**	10.61 - 11.14	10.61 - 11.31	10.76 - 11.32	13.05 - 13.41	13.08 - 13.45	12.98 - 13.46
**CD70**	7.98 - 8.38	8.14 - 8.53	8.11 - 8.73	8.59 - 9.31	8.50 - 9.55	8.51 - 9.03
**CD80**	4.77 - 6.42	5.12 - 7.03	6.05 - 6.73	12.50 - 12.95	12.26 - 13.29	12.68 - 12.92
**CD83**	14.17 - 15.11	14.07 - 15.22	14.37 - 15.35	12.82 - 13.31	12.86 - 13.54	12.75 - 13.40
**CD93**	0.00 - 5.59	0.00 - 6.32	4.44 - 6.23	13.21 - 13.68	13.11 - 13.81	13.10 - 13.80
**CEACAM5**	5.88 - 6.86	6.17 - 6.93	6.36 - 7.11	12.56 - 13.65	12.31 - 13.91	12.64 - 13.89
**CHI3L1**	12.86 - 14.14	13.35 - 14.75	14.03 - 15.16	11.10 - 11.91	11.36 - 12.33	11.12 - 12.60
**CLEC4A**	9.43 - 10.06	9.65 - 10.29	9.76 - 10.42	13.52 - 13.97	13.58 - 14.42	13.20 - 13.87
**CNTF**	6.30 - 6.82	6.59 - 6.93	6.46 - 6.83	7.29 - 8.86	7.69 - 10.30	7.41 - 9.72
**CRP**	9.44 - 11.68	9.43 - 12.55	9.91 - 11.82	9.49 - 11.52	10.23 - 11.16	10.08 - 11.10
**CSF1**	11.31 - 12.05	11.20 - 12.08	11.28 - 12.22	12.02 - 12.57	12.27 - 12.86	12.15 - 12.66
**CSF1R**	6.23 - 7.72	5.98 - 8.02	5.98 - 8.42	12.38 - 13.27	12.60 - 13.76	12.37 - 13.30
**CSF2**	9.33 - 10.11	9.45 - 11.06	9.63 - 10.62	12.01 - 12.94	12.11 - 13.01	11.89 - 12.74
**CSF2RB**	6.12 - 7.52	6.73 - 7.99	7.00 - 7.92	12.12 - 13.88	13.62 - 14.41	12.22 - 13.93
**CSF3**	8.41 - 8.98	8.33 - 9.30	8.26 - 9.38	12.54 - 13.43	12.74 - 13.99	12.50 - 12.91
**CSF3R**	6.04 - 7.17	6.56 - 7.23	6.46 - 7.70	12.61 - 13.10	12.75 - 13.34	12.82 - 13.16
**CST7**	7.12 - 8.52	7.44 - 8.79	7.31 - 8.97	12.55 - 13.34	12.83 - 14.05	12.32 - 12.99
**CTF1**	7.18 - 8.88	6.33 - 7.89	6.82 - 7.92	12.17 - 14.64	11.70 - 14.10	11.42 - 15.08
**CTLA4**	9.88 - 11.08	10.50 - 11.89	10.49 - 11.94	12.93 - 13.47	12.94 - 13.78	13.02 - 13.60
**CTSS**	3.11 - 13.06	0.00 - 12.83	0.00 - 12.77	1.14 - 14.40	0.00 - 15.03	1.32 - 14.82
**CX3CL1**	10.40 - 11.42	10.40 - 11.36	10.44 - 11.27	13.09 - 13.67	13.25 - 13.97	13.08 - 13.97
**CXADR**	14.78 - 15.62	14.47 - 15.59	14.50 - 15.58	12.32 - 13.07	12.56 - 13.28	12.42 - 13.24
**CXCL1**	10.54 - 11.19	10.83 - 11.92	11.14 - 11.65	11.56 - 12.96	11.21 - 12.80	11.61 - 13.01
**CXCL10**	11.51 - 12.98	12.34 - 14.11	12.43 - 13.98	11.89 - 12.66	11.39 - 13.37	12.04 - 13.22
**CXCL11**	3.28 - 7.13	5.97 - 8.27	6.10 - 8.34	11.08 - 13.00	10.16 - 12.79	11.40 - 12.64
**CXCL12**	8.95 - 9.19	8.94 - 9.20	9.06 - 9.19	9.86 - 12.28	10.38 - 12.90	9.95 - 12.44
**CXCL13**	6.36 - 8.08	8.35 - 12.26	8.09 - 11.13	12.77 - 13.46	12.52 - 13.29	12.40 - 13.12
**CXCL14**	10.47 - 10.65	10.55 - 10.77	10.54 - 10.75	10.68 - 12.88	10.80 - 11.94	10.67 - 13.26
**CXCL16**	12.78 - 13.60	12.62 - 13.66	13.10 - 14.08	12.42 - 13.08	12.65 - 13.07	12.78 - 13.30
**CXCL2**	5.31 - 6.94	5.50 - 6.99	6.06 - 7.11	11.97 - 13.79	11.33 - 13.59	11.26 - 13.65
**CXCL3**	6.20 - 7.35	6.61 - 7.76	6.89 - 7.57	12.36 - 14.05	11.87 - 13.55	12.00 - 14.11
**CXCL5**	5.69 - 6.78	5.83 - 7.13	5.66 - 7.40	11.53 - 13.43	11.26 - 13.37	10.85 - 13.21
**CXCL6**	9.59 - 10.00	9.55 - 10.09	9.86 - 10.31	9.62 - 10.42	9.75 - 10.33	9.65 - 10.41
**CXCL8**	7.64 - 8.53	7.76 - 8.82	7.93 - 8.91	5.44 - 5.99	5.24 - 5.74	5.44 - 6.04
**CXCL9**	6.75 - 8.54	7.61 - 9.20	7.61 - 9.35	11.91 - 12.87	11.27 - 13.59	12.05 - 13.16
**EGF**	0.00 - 4.42	0.00 - 4.11	0.00 - 4.53	11.06 - 13.77	11.13 - 13.28	10.92 - 13.44
**EPO**	9.50 - 9.93	9.29 - 9.77	9.56 - 10.09	12.30 - 13.14	12.24 - 13.20	12.28 - 13.22
**FASLG**	9.16 - 10.29	9.54 - 10.80	9.73 - 10.86	13.25 - 13.83	12.96 - 14.21	12.93 - 13.62

**FGF19**	6.28 - 7.99	5.41 - 7.75	7.06 - 8.32	12.56 - 13.62	12.04 - 13.73	12.21 - 13.91
**FGF2**	9.90 - 10.65	10.18 - 10.64	10.08 - 10.85	12.52 - 14.65	12.47 - 13.79	11.80 - 14.66
**FGF21**	5.16 - 6.75	5.24 - 6.54	5.84 - 7.49	9.27 - 11.44	10.00 - 11.23	10.22 - 11.78
**FGF23**	8.29 - 9.23	8.44 - 9.13	8.41 - 9.62	11.50 - 12.15	11.22 - 12.30	11.25 - 12.19
**FLT1**	11.52 - 12.22	11.36 - 12.58	11.41 - 12.14	13.27 - 13.77	13.50 - 13.98	13.22 - 13.90
**FLT3LG**	6.41 - 7.47	6.72 - 7.68	6.78 - 8.18	7.75 - 8.60	7.90 - 8.84	8.07 - 8.89
**FLT4**	4.07 - 5.18	3.71 - 5.38	4.05 - 5.47	12.75 - 13.30	12.95 - 13.48	12.89 - 13.33
**FTH1**	11.68 - 12.47	11.15 - 12.47	11.65 - 12.49	13.10 - 14.00	13.25 - 14.50	13.95 - 14.60
**FURIN**	6.71 - 7.32	6.77 - 7.54	7.02 - 7.98	11.96 - 12.88	12.01 - 13.40	12.10 - 13.28
**GDF15**	8.70 - 9.84	9.08 - 10.37	8.98 - 10.74	12.42 - 13.65	12.50 - 13.79	12.19 - 13.88
**GDF2**	6.90 - 8.24	7.05 - 7.84	7.23 - 8.12	11.40 - 12.47	11.03 - 12.42	11.14 - 12.72
**GFAP**	19.11 - 20.24	19.24 - 20.39	19.87 - 20.75	13.41 - 14.10	13.25 - 14.30	13.45 - 14.62
**GRN**	6.95 - 7.91	7.04 - 8.08	6.11 - 8.08	12.69 - 13.18	12.53 - 13.37	12.39 - 13.24
**GZMA**	9.84 - 10.96	10.54 - 11.88	10.53 - 11.79	12.55 - 12.97	12.23 - 13.29	12.37 - 12.85
**GZMB**	2.82 - 4.88	4.23 - 5.74	3.06 - 5.46	8.93 - 10.49	9.22 - 11.35	9.40 - 10.80
**HAVCR1**	3.97 - 6.16	4.73 - 5.95	3.99 - 5.99	11.29 - 12.97	11.72 - 12.78	12.41 - 13.16
**HGF**	13.93 - 14.88	13.79 - 15.01	14.16 - 14.96	13.03 - 13.68	13.29 - 14.19	13.19 - 13.75
**HLADRA**	11.45 - 12.71	11.57 - 12.60	12.11 - 13.05	12.15 - 13.11	12.14 - 13.25	12.04 - 13.31
**ICAM1**	4.30 - 5.95	3.70 - 5.54	4.52 - 5.87	12.46 - 13.03	12.74 - 13.14	12.58 - 13.28
**ICOSLG**	5.68 - 7.12	5.61 - 7.06	6.00 - 7.23	12.74 - 13.24	12.78 - 13.09	12.73 - 13.32
**IFNA1__IFNA13**	9.47 - 10.62	9.44 - 10.65	9.75 - 10.78	7.93 - 8.99	8.01 - 9.13	7.94 - 9.97
**IFNA2**	8.28 - 8.74	8.34 - 8.88	8.30 - 8.93	8.62 - 9.31	8.66 - 9.61	8.63 - 9.63
**IFNB1**	9.56 - 10.36	9.72 - 10.39	9.96 - 10.96	12.27 - 13.93	12.09 - 13.82	12.29 - 14.62
**IFNG**	5.34 - 6.76	6.35 - 9.48	5.87 - 7.98	10.34 - 11.49	10.00 - 11.37	10.47 - 11.88
**IFNL1**	10.38 - 11.20	10.44 - 11.47	10.72 - 11.64	13.71 - 14.72	13.85 - 15.16	13.64 - 14.99
**IFNL2__IFNL3**	2.64 - 3.02	2.80 - 3.31	2.75 - 3.22	4.52 - 5.17	4.13 - 5.69	4.28 - 6.01
**IFNW1**	9.04 - 10.04	8.99 - 10.14	9.03 - 10.40	9.23 - 10.06	8.83 - 9.87	9.20 - 10.11
**IKBKG**	4.63 - 6.01	3.93 - 5.97	4.44 - 5.86	11.43 - 13.64	11.48 - 12.76	11.06 - 12.82
**IL10**	8.04 - 9.38	9.07 - 10.71	9.07 - 10.52	10.78 - 11.84	11.18 - 12.04	10.80 - 12.12
**IL10RB**	8.38 - 9.14	8.37 - 9.40	8.52 - 9.61	12.88 - 13.21	12.83 - 13.47	12.94 - 13.50
**IL11**	6.86 - 7.49	6.90 - 7.44	7.01 - 7.52	7.86 - 8.92	7.54 - 9.01	7.42 - 8.98
**IL12B**	6.76 - 7.88	7.76 - 9.70	7.56 - 9.24	12.39 - 13.13	12.63 - 13.69	12.14 - 13.30
**IL12RB1**	7.94 - 9.11	7.79 - 9.42	8.28 - 9.25	12.74 - 13.24	12.61 - 13.40	12.71 - 13.28
**IL12p70**	8.40 - 9.35	8.74 - 10.31	8.80 - 9.98	12.63 - 13.62	12.61 - 14.22	12.97 - 13.45
**IL13**	9.89 - 11.05	9.71 - 11.03	9.97 - 11.23	12.92 - 14.19	12.87 - 13.88	12.67 - 14.08
**IL13RA2**	8.99 - 9.78	8.80 - 9.72	9.08 - 9.88	9.89 - 10.25	9.56 - 10.03	9.94 - 10.17
**IL15**	12.26 - 13.06	12.01 - 13.36	12.61 - 13.27	12.73 - 13.17	13.04 - 13.41	12.90 - 13.28
**IL15RA**	8.14 - 9.46	8.41 - 9.95	8.47 - 9.57	12.75 - 13.32	12.82 - 13.81	12.86 - 13.51
**IL16**	7.30 - 8.26	7.33 - 8.31	7.30 - 8.18	12.57 - 13.00	12.64 - 13.25	12.40 - 13.07
**IL17A**	5.91 - 6.23	6.02 - 6.52	6.02 - 6.57	7.59 - 8.57	7.25 - 9.53	7.39 - 8.25
**IL17A_IL17F**	3.35 - 4.62	3.61 - 4.71	3.58 - 5.01	6.27 - 7.16	5.62 - 7.40	5.80 - 7.09
**IL17B**	16.75 - 17.64	16.60 - 18.00	16.79 - 17.95	13.57 - 14.33	13.07 - 14.16	13.26 - 13.98
**IL17C**	5.12 - 6.73	4.59 - 6.97	5.73 - 7.12	12.71 - 13.79	12.44 - 13.76	12.53 - 13.02

**IL17F**	8.75 - 9.01	8.94 - 9.13	8.93 - 9.09	10.17 - 12.10	10.31 - 12.25	9.59 - 10.86
**IL17RA**	8.31 - 9.27	8.36 - 9.38	8.62 - 9.58	13.09 - 13.32	13.06 - 13.55	13.12 - 13.46
**IL17RB**	9.96 - 11.14	9.90 - 11.01	9.90 - 10.97	12.81 - 14.06	13.69 - 14.77	13.04 - 14.90
**IL18**	7.50 - 8.96	6.66 - 8.31	6.96 - 8.79	11.76 - 13.76	11.88 - 12.96	11.73 - 13.46
**IL18BP**	10.29 - 11.34	10.29 - 11.57	10.73 - 11.76	12.75 - 13.12	12.75 - 13.45	12.90 - 13.23
**IL18R1**	6.34 - 7.65	6.52 - 8.04	7.06 - 8.39	12.47 - 13.11	12.68 - 13.29	12.80 - 13.16
**IL19**	8.98 - 10.40	8.84 - 10.50	9.09 - 10.17	12.50 - 13.25	12.43 - 14.26	12.05 - 13.20
**IL1B**	6.77 - 7.13	7.08 - 7.35	7.13 - 7.40	7.60 - 8.46	7.47 - 8.19	7.37 - 8.36
**IL1R1**	5.74 - 7.04	5.44 - 7.15	6.07 - 7.35	13.32 - 13.68	13.42 - 13.82	13.23 - 13.74
**IL1R2**	0.00 - 5.42	0.00 - 5.83	0.00 - 5.80	12.87 - 13.34	12.87 - 13.26	12.81 - 13.30
**IL1RL1**	0.00 - 5.93	0.00 - 6.45	0.00 - 6.69	12.30 - 13.45	12.40 - 13.44	12.80 - 13.31
**IL1RN**	8.12 - 9.25	8.86 - 9.90	8.42 - 9.49	11.20 - 11.84	11.79 - 12.48	11.25 - 12.01
**IL2**	4.38 - 5.42	4.82 - 6.16	4.91 - 6.02	9.25 - 10.01	8.95 - 9.83	9.21 - 10.08
**IL20**	11.04 - 11.71	10.87 - 11.71	10.81 - 11.77	12.86 - 13.40	12.99 - 13.41	12.73 - 13.22
**IL22**	10.02 - 10.99	10.05 - 10.69	10.22 - 10.97	11.66 - 13.66	11.15 - 12.88	11.80 - 13.39
**IL23**	11.58 - 12.39	12.01 - 13.23	12.07 - 13.07	13.46 - 14.26	13.73 - 14.74	13.47 - 14.50
**IL24**	8.56 - 9.12	8.51 - 8.98	8.57 - 9.05	9.15 - 10.36	9.04 - 10.99	9.45 - 10.50
**IL27**	7.67 - 8.97	8.00 - 8.91	7.85 - 9.11	12.44 - 13.81	12.75 - 13.86	10.69 - 13.64
**IL2RA**	4.69 - 6.40	4.82 - 6.79	4.24 - 7.25	12.74 - 13.18	12.77 - 13.40	12.50 - 13.33
**IL2RB**	6.38 - 7.84	6.67 - 8.19	7.05 - 8.14	12.94 - 13.48	12.89 - 13.64	12.75 - 13.29
**IL32**	7.15 - 7.49	7.29 - 7.55	7.30 - 7.56	7.25 - 8.68	7.25 - 7.59	7.26 - 7.82
**IL33**	6.49 - 7.05	6.62 - 7.41	6.66 - 7.35	8.77 - 9.52	8.65 - 9.32	8.65 - 9.21
**IL34**	15.25 - 16.24	14.88 - 16.30	14.92 - 16.39	12.31 - 13.02	12.37 - 13.47	11.91 - 12.91
**IL36A**	5.67 - 7.94	4.95 - 7.82	5.77 - 8.13	13.37 - 13.99	13.08 - 14.11	12.98 - 13.97
**IL36B**	5.50 - 6.39	6.99 - 7.34	5.98 - 7.39	8.04 - 8.94	8.11 - 8.41	7.82 - 8.72
**IL36G**	5.16 - 5.70	5.27 - 5.60	5.24 - 5.78	6.90 - 7.23	6.36 - 7.01	6.70 - 7.10
**IL3RA**	8.64 - 9.75	8.78 - 9.99	8.57 - 9.99	12.86 - 13.50	13.15 - 13.61	13.03 - 13.74
**IL4**	6.21 - 7.47	6.44 - 7.75	6.29 - 8.02	8.60 - 9.41	8.50 - 9.35	8.44 - 9.31
**IL4R**	6.44 - 8.22	6.34 - 8.10	6.58 - 8.29	13.31 - 13.55	13.23 - 13.76	13.33 - 13.64
**IL5**	12.59 - 13.69	12.44 - 13.53	12.56 - 13.63	12.58 - 13.79	12.10 - 13.29	12.34 - 14.02
**IL5RA**	0.00 - 5.98	0.00 - 6.97	5.58 - 7.52	12.18 - 13.23	12.49 - 13.64	12.86 - 13.42
**IL6**	11.60 - 12.38	11.46 - 12.80	11.70 - 12.64	10.97 - 12.44	10.96 - 12.59	11.46 - 12.20
**IL6R**	6.94 - 8.31	6.99 - 8.54	7.15 - 8.75	12.81 - 13.50	13.13 - 13.52	12.82 - 13.48
**IL6ST**	10.00 - 10.86	9.66 - 10.85	9.98 - 10.98	13.21 - 13.49	13.35 - 13.78	13.25 - 13.75
**IL7**	9.66 - 10.41	9.01 - 10.37	9.52 - 10.50	12.13 - 13.33	11.94 - 13.70	12.32 - 13.30
**IL7R**	3.61 - 5.25	3.54 - 5.29	3.32 - 5.13	13.05 - 13.86	12.94 - 14.06	13.11 - 13.78
**IL9**	14.05 - 15.02	13.87 - 15.28	14.09 - 14.95	12.74 - 13.57	12.78 - 13.87	12.50 - 13.53
**IRAK4**	9.46 - 9.89	9.71 - 10.09	9.52 - 10.03	13.10 - 16.69	12.16 - 14.82	12.06 - 16.44
**KDR**	6.65 - 7.70	6.37 - 7.77	6.66 - 8.35	12.67 - 13.37	12.92 - 13.85	13.05 - 13.69
**KITLG**	7.69 - 7.95	8.06 - 8.29	7.97 - 8.37	9.57 - 10.07	9.57 - 9.98	9.62 - 10.15
**KLRK1**	7.53 - 8.66	7.61 - 8.71	7.88 - 9.00	12.97 - 13.35	12.71 - 13.60	12.83 - 13.44
**KNG1**	5.31 - 5.59	5.50 - 5.83	5.40 - 5.69	12.88 - 13.42	13.23 - 13.70	12.97 - 13.77
**LAG3**	5.60 - 7.38	6.58 - 8.03	7.01 - 8.52	12.52 - 13.18	12.84 - 13.46	12.67 - 13.37

**LAMP3**	8.65 - 9.11	8.79 - 9.10	8.66 - 9.06	10.74 - 11.51	10.79 - 11.22	10.76 - 11.76
**LCN2**	4.98 - 7.50	4.83 - 7.52	5.70 - 7.65	13.31 - 13.80	13.49 - 14.01	13.39 - 13.91
**LGALS9**	9.29 - 10.24	9.30 - 10.54	9.66 - 10.55	12.58 - 13.11	12.69 - 13.27	12.56 - 13.05
**LIF**	11.77 - 12.93	11.60 - 12.89	11.83 - 12.94	8.75 - 9.26	8.85 - 9.93	8.71 - 9.91
**LILRB2**	4.59 - 6.43	4.90 - 6.65	5.52 - 7.06	12.70 - 13.42	12.99 - 13.58	12.86 - 13.54
**LTA**	7.34 - 7.77	7.56 - 8.28	7.43 - 8.08	8.42 - 8.90	8.39 - 8.74	8.37 - 8.71
**LTA_LTB**	7.03 - 7.38	7.14 - 7.48	7.16 - 7.56	8.90 - 11.40	9.48 - 11.86	9.16 - 11.92
**MERTK**	8.15 - 8.85	8.15 - 9.39	8.15 - 9.28	12.93 - 13.39	13.01 - 13.62	13.03 - 13.54
**MICA**	9.38 - 11.01	10.05 - 10.95	10.12 - 11.20	9.76 - 13.88	12.24 - 14.27	12.49 - 14.51
**MICB**	8.84 - 11.17	8.89 - 10.97	8.92 - 11.01	11.95 - 12.94	12.04 - 13.06	11.47 - 13.09
**MIF**	6.57 - 8.17	6.54 - 8.48	6.57 - 9.09	11.83 - 14.84	11.78 - 13.49	12.00 - 14.43
**MMP1**	6.81 - 8.20	7.05 - 8.04	7.04 - 8.50	11.98 - 13.90	12.18 - 14.35	11.79 - 13.52
**MMP12**	6.58 - 7.75	6.64 - 7.98	7.05 - 8.84	13.16 - 13.85	12.83 - 13.71	12.90 - 14.15
**MMP3**	6.66 - 8.12	6.65 - 8.32	6.93 - 8.14	13.11 - 14.11	12.78 - 14.38	13.37 - 14.26
**MMP8**	1.78 - 5.23	3.03 - 5.48	3.65 - 5.64	12.45 - 13.22	13.27 - 14.12	12.99 - 13.36
**MMP9**	0.00 - 2.12	0.00 - 3.48	0.00 - 0.91	13.19 - 14.77	13.96 - 15.80	13.28 - 15.03
**MPO**	3.89 - 5.92	4.15 - 5.72	4.08 - 6.40	11.53 - 12.37	12.33 - 12.97	11.46 - 12.29
**MUC16**	4.48 - 5.86	4.91 - 6.23	4.11 - 6.01	12.34 - 13.41	12.60 - 13.49	12.26 - 13.49
**NAMPT**	0.00 - 8.50	0.00 - 9.01	0.00 - 9.40	10.50 - 11.66	11.11 - 12.03	10.77 - 11.76
**NCR1**	4.33 - 6.68	5.43 - 7.51	5.72 - 7.29	12.81 - 13.35	12.85 - 13.57	12.95 - 13.59
**NGF**	8.93 - 9.38	9.00 - 9.46	9.01 - 9.53	11.48 - 11.94	11.59 - 12.22	11.37 - 11.80
**NTF3**	8.68 - 8.94	8.75 - 8.96	8.72 - 8.92	10.18 - 12.18	10.29 - 11.38	10.35 - 12.69
**OSM**	8.62 - 9.37	8.81 - 9.77	8.77 - 9.48	13.62 - 14.85	14.43 - 15.74	13.93 - 14.85
**OSMR**	7.22 - 8.33	7.01 - 8.27	7.22 - 8.76	12.68 - 13.19	12.76 - 13.37	12.74 - 13.24
**PDCD1**	6.87 - 8.14	7.49 - 8.59	7.40 - 8.77	12.61 - 13.13	12.85 - 13.61	12.65 - 13.04
**PDCD1LG2**	5.50 - 6.94	5.50 - 7.35	6.38 - 7.29	12.98 - 13.42	13.22 - 13.73	13.12 - 13.70
**PDGFA**	9.92 - 10.20	10.16 - 10.43	10.04 - 10.38	12.94 - 14.20	12.63 - 14.21	12.69 - 13.93
**PDGFB**	0.00 - 0.00	0.00 - 0.00	0.00 - 0.00	12.74 - 14.43	12.31 - 14.22	12.60 - 14.09
**PGF**	13.52 - 14.38	13.54 - 14.57	13.96 - 14.97	13.10 - 13.54	13.24 - 13.81	13.43 - 13.83
**PTX3**	3.28 - 6.39	0.00 - 6.21	4.64 - 6.64	12.86 - 13.75	13.26 - 13.85	12.64 - 13.57
**S100A12**	3.28 - 4.75	3.50 - 4.82	2.69 - 4.58	11.66 - 13.25	12.60 - 14.10	12.00 - 12.76
**S100A9**	8.60 - 9.16	9.23 - 9.56	8.89 - 9.50	12.83 - 14.07	12.73 - 13.96	12.33 - 13.69
**SCG2**	11.85 - 13.30	11.67 - 13.32	12.13 - 13.56	12.14 - 13.61	12.12 - 13.60	10.82 - 13.75
**SDC1**	8.00 - 9.07	8.18 - 9.50	8.61 - 9.37	12.65 - 13.28	12.82 - 13.24	12.56 - 13.21
**SELE**	2.81 - 4.82	1.67 - 4.36	2.39 - 4.58	11.97 - 12.97	12.24 - 13.08	12.19 - 12.85
**SELP**	0.00 - 0.00	0.00 - 0.00	0.00 - 3.23	12.32 - 13.17	12.50 - 13.58	12.54 - 13.07
**SIRPA**	13.76 - 14.67	13.37 - 14.73	13.83 - 14.92	12.67 - 13.29	12.97 - 13.40	13.12 - 13.53
**SLAMF1**	3.91 - 6.47	5.17 - 7.30	5.18 - 7.65	12.53 - 13.02	12.47 - 13.07	12.49 - 13.09
**SPP1**	14.67 - 16.13	15.05 - 16.21	15.76 - 16.50	13.08 - 14.00	12.94 - 14.39	13.02 - 14.41
**TAFA5**	21.78 - 23.39	21.48 - 23.63	21.75 - 23.58	13.54 - 14.28	13.57 - 14.53	13.39 - 14.24
**TEK**	5.43 - 6.85	5.19 - 7.11	6.02 - 7.06	12.95 - 13.45	13.04 - 13.64	12.98 - 13.55
**TGFB1**	12.70 - 13.12	12.71 - 13.20	12.94 - 13.34	12.98 - 13.31	12.87 - 13.27	12.95 - 13.26
**TGFB3**	11.89 - 12.84	11.93 - 12.49	11.89 - 12.62	11.45 - 12.04	11.68 - 12.73	11.55 - 12.26

**THBS2**	12.22 - 12.85	11.91 - 12.65	12.26 - 13.05	12.38 - 12.96	12.25 - 12.89	12.24 - 12.86
**THPO**	5.47 - 6.84	4.86 - 6.83	4.82 - 6.81	13.12 - 13.52	13.10 - 13.77	13.10 - 13.51
**TIMP1**	10.07 - 11.47	10.42 - 11.96	10.67 - 11.86	12.62 - 13.41	12.57 - 13.57	12.49 - 13.54
**TIMP2**	10.42 - 11.30	10.14 - 11.20	10.51 - 11.72	10.05 - 10.37	10.14 - 10.39	10.00 - 10.39
**TLR3**	11.84 - 12.51	11.32 - 12.78	11.98 - 12.89	12.10 - 13.20	12.64 - 13.30	12.39 - 13.12
**TNF**	9.82 - 10.77	10.14 - 10.94	9.95 - 11.08	12.72 - 13.06	12.94 - 13.49	12.69 - 13.26
**TNFRSF11A**	12.25 - 13.11	12.23 - 13.30	12.22 - 13.61	12.67 - 13.15	12.77 - 13.24	12.55 - 13.16
**TNFRSF11B**	11.49 - 12.51	11.57 - 12.56	11.84 - 12.93	12.73 - 13.16	12.68 - 13.23	12.71 - 13.32
**TNFRSF13B**	6.87 - 8.00	8.99 - 11.53	9.04 - 11.51	12.73 - 13.28	12.49 - 13.32	12.48 - 13.21
**TNFRSF13C**	15.20 - 16.03	14.97 - 16.55	14.92 - 16.54	12.85 - 13.40	12.59 - 13.44	12.00 - 12.99
**TNFRSF14**	11.02 - 11.87	11.06 - 12.15	11.29 - 12.24	12.83 - 13.28	13.03 - 13.46	12.90 - 13.55
**TNFRSF17**	0.00 - 6.52	6.25 - 7.66	4.44 - 7.82	13.04 - 13.81	13.31 - 13.97	13.08 - 13.82
**TNFRSF18**	14.80 - 16.00	14.74 - 16.06	14.64 - 15.52	13.54 - 13.97	13.41 - 13.78	13.65 - 13.92
**TNFRSF1A**	11.25 - 12.08	11.25 - 12.28	11.53 - 12.66	12.79 - 13.18	12.86 - 13.16	12.63 - 13.10
**TNFRSF1B**	8.68 - 9.37	8.88 - 9.99	9.12 - 10.45	12.47 - 12.90	12.61 - 13.06	12.35 - 13.12
**TNFRSF21**	14.07 - 15.70	13.73 - 15.91	13.87 - 15.72	11.99 - 12.41	12.01 - 12.44	11.95 - 12.60
**TNFRSF4**	8.88 - 9.68	8.81 - 10.16	8.99 - 10.17	13.13 - 13.64	13.06 - 13.76	13.12 - 13.83
**TNFRSF8**	10.53 - 11.64	10.70 - 11.77	10.93 - 12.06	12.48 - 12.99	12.51 - 13.39	12.51 - 12.99
**TNFRSF9**	8.43 - 8.83	8.73 - 9.06	8.76 - 9.05	10.86 - 11.16	10.71 - 11.29	10.67 - 11.15
**TNFSF10**	6.39 - 8.10	5.84 - 8.07	6.26 - 8.34	12.96 - 13.45	13.25 - 13.82	13.23 - 13.78
**TNFSF11**	7.13 - 7.61	7.06 - 7.81	7.13 - 7.70	12.73 - 13.97	13.09 - 14.03	12.31 - 13.86
**TNFSF12**	14.49 - 15.35	14.27 - 15.38	14.56 - 15.42	13.44 - 13.93	13.45 - 14.19	13.60 - 13.91
**TNFSF13**	11.22 - 12.14	10.42 - 12.09	11.04 - 11.95	13.05 - 14.17	12.92 - 14.52	13.16 - 14.13
**TNFSF14**	3.21 - 5.04	4.15 - 5.33	4.16 - 5.67	11.47 - 12.61	12.01 - 12.81	11.96 - 12.48
**TNFSF15**	10.16 - 11.17	10.13 - 11.29	10.23 - 11.36	13.34 - 13.97	13.43 - 14.01	13.53 - 14.05
**TNFSF18**	14.37 - 15.20	14.48 - 15.50	14.34 - 15.50	12.89 - 13.34	12.86 - 13.56	12.90 - 13.41
**TNFSF4**	9.03 - 9.93	9.17 - 10.01	9.24 - 10.16	13.02 - 13.42	13.05 - 13.53	12.91 - 13.48
**TNFSF8**	11.52 - 12.39	11.58 - 12.79	11.96 - 12.94	12.94 - 13.48	13.08 - 13.67	13.11 - 13.55
**TNFSF9**	7.86 - 8.27	7.98 - 8.46	7.81 - 8.41	10.10 - 10.51	10.03 - 10.54	10.15 - 10.37
**TREM1**	7.39 - 8.96	8.10 - 9.64	8.64 - 10.48	12.86 - 13.52	13.39 - 13.81	12.84 - 13.54
**TREM2**	11.92 - 12.83	12.25 - 13.15	12.54 - 13.63	10.95 - 12.08	11.29 - 12.19	11.18 - 12.37
**TSLP**	11.45 - 12.31	11.30 - 12.11	11.68 - 12.70	11.72 - 12.61	12.14 - 12.70	12.20 - 12.86
**VCAM1**	8.20 - 9.24	8.25 - 9.26	8.43 - 9.43	13.16 - 13.48	12.94 - 13.73	13.14 - 13.54
**VEGFA**	14.71 - 15.43	14.36 - 15.41	14.43 - 15.42	13.88 - 14.24	13.78 - 14.20	13.87 - 14.29
**VEGFC**	11.47 - 12.13	11.75 - 12.42	11.81 - 12.29	13.25 - 13.69	13.29 - 13.65	13.17 - 13.67
**VEGFD**	5.87 - 6.41	6.28 - 6.70	6.16 - 6.68	6.88 - 7.97	6.72 - 7.80	6.81 - 8.86
**VSNL1**	12.68 - 13.69	12.37 - 13.80	12.57 - 13.39	11.98 - 12.42	11.79 - 12.57	11.88 - 12.28
**VSTM1**	5.81 - 7.49	6.06 - 7.49	6.07 - 7.79	12.49 - 13.97	12.99 - 14.41	12.91 - 13.62
**WNT16**	14.23 - 15.28	14.13 - 15.24	14.31 - 15.39	13.27 - 14.10	12.98 - 14.46	13.51 - 13.90
**WNT7A**	9.27 - 10.21	9.08 - 10.40	9.13 - 10.11	9.53 - 10.16	9.40 - 10.36	9.43 - 10.27
